# Inner Filter Effect Correction for Fluorescence Measurements in Microplates Using Variable Vertical Axis Focus

**DOI:** 10.1021/acs.analchem.2c01031

**Published:** 2022-05-03

**Authors:** Tin Weitner, Tomislav Friganović, Davor Šakić

**Affiliations:** Faculty of Pharmacy and Biochemistry, University of Zagreb, Ante Kovačića 1, Zagreb 10000, Croatia

## Abstract

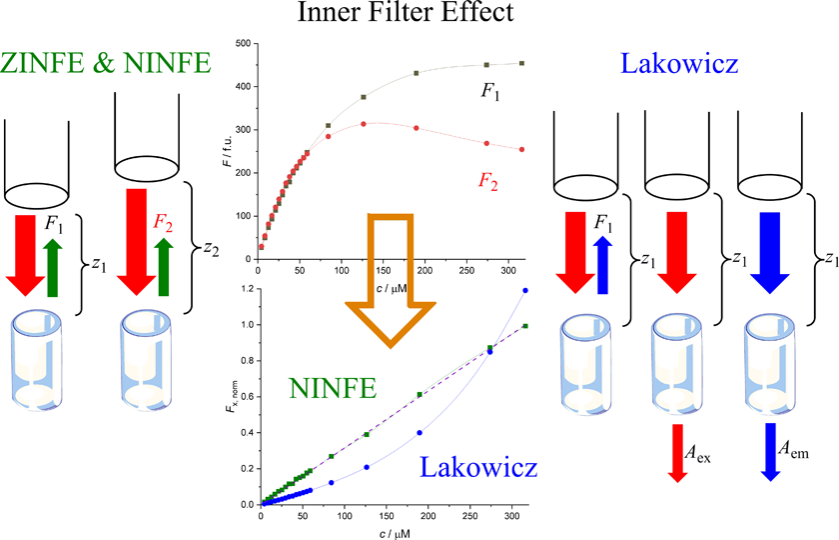

The inner filter effect (IFE) hinders fluorescence measurements, limiting linear dependence of fluorescence signals to low sample concentrations. Modern microplate readers allow movement of the optical element in the vertical axis, changing the relative position of the focus and thus the sample geometry. The proposed *Z*-position IFE correction method requires only two fluorescence measurements at different known vertical axis positions (*z*-positions) of the optical element for the same sample. Samples of quinine sulfate, both pure and in mixtures with potassium dichromate, showed a linear dependence of corrected fluorescence on fluorophore concentration (*R*^2^ > 0.999), up to *A*_ex_ ≈ 2 and *A*_em_ ≈ 0.5. The correction extended linear fluorescence response over ≈98% of the concentration range with ≈1% deviation of the calibration slope, effectively eliminating the need for sample dilution or separate absorbance measurements to account for IFE. The companion numerical IFE correction method further eliminates the need for any geometric parameters with similar results. Both methods are available online at https://ninfe.science.

## Introduction

### Inner Filter Effect in Fluorescence Spectroscopy

Fluorescence has proven to be an outstanding tool for investigating the structure and dynamics of matter or living systems, with applications in the physical, chemical, material, biological, and medical sciences.^1^ Advances in fluorescence technology have resulted in reduction of the cost and complexity of measurement instruments, and fluorescence spectroscopy will continue to contribute to rapid advances in biology, biotechnology, and nanotechnology.^2^ Currently, fluorescence experiments for binding studies, quenching, and cell-based assays are being designed using microplate readers that allow the acquisition of spectra, anisotropies, and lifetimes.^2^ The optics used in microplate readers is different from those of an instrument designed for use with a cuvette. Typically, the microplate is moved using an *x*–*y* scanning stage to position each well in the observation path.^2^

As has been noted by many authors, the apparent fluorescence intensity and spectral distribution can depend on the optical density of the sample and the precise geometry of the sample illumination.^2−4^ These effects can (i) reduce the intensity of the excitation at the observation point or (ii) reduce the observed fluorescence by absorbing the emitted fluorescence.^2^ The resulting influences of (i) and (ii) on the detected signal are known as primary inner filter effect (IFE) and secondary IFE, or pIFE and sIFE, respectively.^3^ The relative importance of each process depends on the optical densities of the sample at the excitation and emission wavelengths.^2^ Therefore, fluorescence intensities are proportional to concentration only in a limited range of optical densities, and the nonlinear dependence of fluorescence intensity on the concentration of the fluorescent substance greatly complicates the determination of parameters derived from fluorescence data.^2,5^ In addition, sIFE can occur for some substances with small Stokes shift if the overlap of the absorption spectrum and fluorescence emission spectrum results in the emitted fluorescence being reabsorbed by the sample.^6^

### Conventional Methods for IFE Correction

Extensive research has addressed the minimization or correction of IFE using mathematical or instrumental procedures, as indicated by a number of recent reviews.^7,8^ In general, the use of dilute solutions is considered the best practice,^2,5^ but it has been shown that IFE correction should also be performed for low fluorophore concentrations.^5^ For example, at an absorbance of *A* = 0.06, the relative error in recorded fluorescence intensity is approximately 8%, and this difference increases further to 12% at *A* = 0.1 and 38% at *A* = 0.3.^5,9^ As previously noted by Wang, sample dilution may introduce additional errors and/or alter the chemical properties of the samples.^8^

A simple and approximate method for IFE correction of observed fluorescence proposed by Lakowicz is shown in eq 1
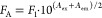
1where *F*_A_ is the absorbance IFE-corrected fluorescence intensity, *F*_1_ is the measured (uncorrected) fluorescence intensity, *A*_ex_ is the absorbance at the fluorescence excitation wavelength, and *A*_em_ is the absorbance at the selected fluorescence emission wavelength.^2^

The main assumption of this method is that the fluorescence light is collected from the center of the cell, which may not be true depending on the geometry of the sample compartment.^5,7^ An additional drawback is that the absorbance of the sample at both λ_ex_ and λ_em_ must be measured independently. For a detailed overview of the properties of this correction method, the article by Panigrahi and Mishra can be referred.^4^ Briefly, the authors described a geometry-dependent maximum of the achievable fluorescence intensity corresponding to a maximum concentration of the analyte, beyond which the observed fluorescence intensity decreases and the emission curve exhibits a downward curvature. They have also shown that the Lakowicz model for the IFE correction is valid only up to *A* = 0.7. For larger values of *A*, this model overestimates the loss of observed fluorescence due to IFE, resulting in an upward curvature of the corrected fluorescence. Notwithstanding its limitations, the Lakowicz model is currently extensively used for correcting IFE-related artifacts in the observed fluorescence intensity.^4,8^ Therefore, this method was chosen as the benchmark for IFE correction.

Another relatively simple option for IFE correction is the cell shift method, in which the fluorescence intensity of the sample is measured at different positions with different effective light path lengths.^8^ This method does not require direct measurements of the sample absorbance at the excitation and emission wavelengths and allows correction for both pIFE and sIFE by measuring the fluorescence intensity at two points in the sample, according to eq 2
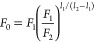
2where *F*_0_ is the corrected fluorescence intensity and *F*_1_ and *F*_2_ are the measured fluorescence values for different light path lengths, *l*_1_ and *l*_2_. When using the cell shift method proposed by Lutz and Luisi, the values of *l*_1_ and *l*_2_ are measured along the diagonal in a standard 1 cm rectangular cell.^10^ However, this method has limited applicability because it requires special instrumentation that is not commonly available, as noted in the literature.^8,11^

### IFE Correction in Microplates

**Figure 1:**
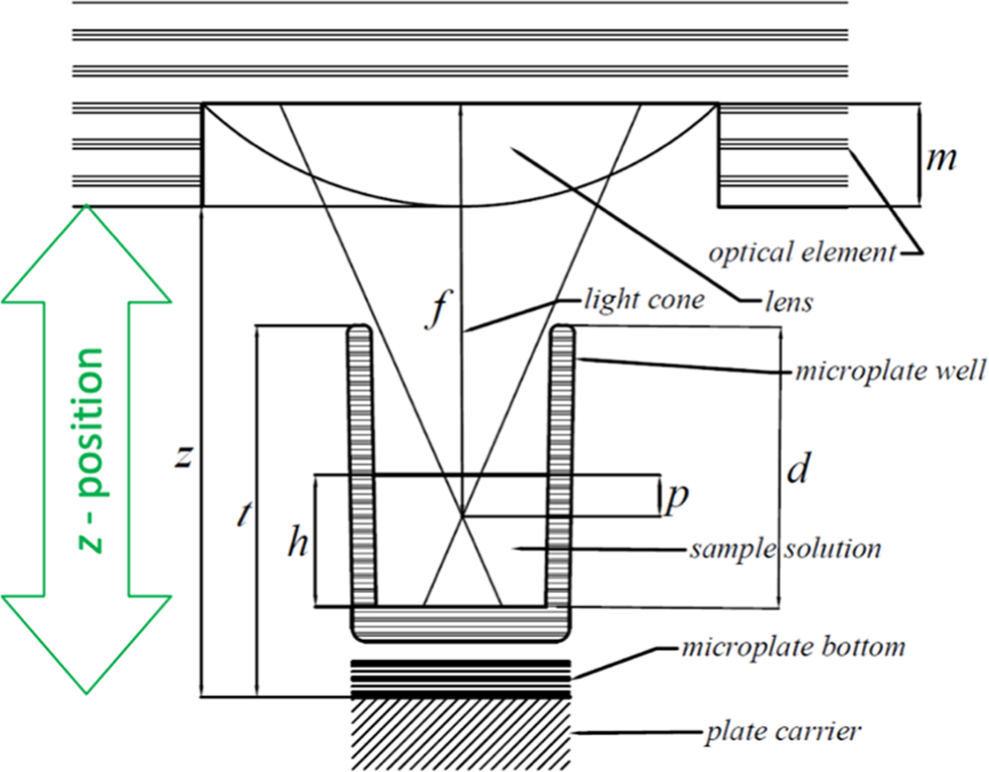
Geometric parameters of the microplate reader used for the ZINFE, eqs 3–5. The values of the parameters used for the calculations can be found in Table S2, Supporting Information.

Unlike a standard cuvette with a fixed light path length, the light path length in a microplate well is unknown and depends on the filling volume of the wells. Modern microplate readers allow the optical element used for excitation and emission to be moved in the *z*-axis (perpendicular to the sample well), allowing the sample geometry to be easily changed with the primary goal of optimizing measurement sensitivity. This movement changes the effective light path lengths, with the geometric parameter *p* corresponding to the distance between the focal point of the measurement and the surface of the liquid in the microplate well. The parameter *p* can be calculated from the known adjustable *z*-position of the optical element and other fixed geometrical parameters of the microplate reader (Figure 1) using eq 3
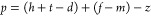
3where *p* is the distance between the focal point of the measurement and the surface of the liquid in the microplate well (corresponding to the parameter *l* in eq 2), *d* is the microplate well depth, *h* is the distance from the bottom of the microplate well to the surface of the liquid, *t* is the total height of the microplate, *f* is the distance from the optical element to the focal point of the lens, *m* is the depth of the lens slot of the optical element, and *z* is the distance from the lens to the bottom of the microplate well (*z*-position).

The parameters *d*, *h*, and *t* are distinctive for different microplate types, whereas the parameter *h* also depends on the sample volume in the well. The parameters *f* and *m* are distinctive for a particular optical system of the microplate reader instrument. A single overall geometric parameter *k* for a particular sample volume, microplate, and microplate reader type can be calculated using eq 4
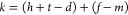
4

The combination of eqs 2 and 4 yields the proposed *Z*-position inner filter effect (ZINFE) correction using eq 5
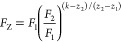
5where *F*_Z_ is the ZINFE-corrected fluorescence intensity, *F*_1_ and *F*_2_ are the measured fluorescence values at different *z*-positions, *z*_1_ and *z*_2_, and *k* is defined in eq 4.

As previously proposed by Lutz and Luisi, eq 5 can be simplified to include a simple exponential term corresponding to a particular combination of *k*, *z*_1_, and *z*_2_. In addition to calculations from geometry-dependent parameters, this exponential term can also be obtained by least-squares fitting from experimental values of *F*_1_ and *F*_2_, thus obtaining the proposed numerical inner filter effect (NINFE) correction using eq 6
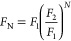
6where *F*_N_ is the NINFE-corrected fluorescence intensity based on fluorescence measurements at different *z*-positions (*F*_1_ and *F*_2_), and the exponential term *N* is obtained by brute-force optimization. This allows a wider range of applicable *z*-positions and also helps to account for possible reflection effects or errors in the estimation of geometric parameters. For such NINFE correction, only two sets of fluorescence data, *F*_1_ and *F*_2_, measured at *z*-positions *z*_1_ and *z*_2_ are needed. The actual values of *z*_1_ and *z*_2_, or indeed any other geometric parameters, are not necessary to obtain the corrected fluorescence, *F*_N_. This correction can also be applied to data generated by the cell shift method mentioned earlier.

### Objective and Limitations

Several recent reports have addressed the IFE correction. Panigrahi and Mishra calculated the geometric parameters from the dependence of measured fluorescence on sample absorbance.^4^ Kasparek and Smyk used horizontal slits in the light path of the spectrofluorometer to numerically optimize the geometric parameters separately for pIFE and sIFE.^12^ Similar to Lutz and Luisi, Kimball *et al.* used a custom stage for lateral cuvette movement in order to determine the geometric sensitivity factor of the spectrofluorometer.^3^ Gu and Kenny also used a custom stage for cell shift experiments with additional numerical optimization of the geometric parameters, also separately for pIFE and sIFE.^13^ However, all these methods are only applicable to conventional spectrofluorometers with detection at a 90° angle in rectangular cuvettes. Moreover, all these methods require separate measurements of sample absorbance and some kind of numerical procedure to account for sample geometry.

The aim of this work is to validate the proposed principle of IFE correction in microplates by comparing uncorrected fluorescence data, *F*_1_, with the values of *F*_Z_, *F*_N_, and *F*_A_ obtained using eqs 5, 6, and 1, respectively. For the first set of experiments, fluorescence and absorbance were measured for the same samples in the same UV-transparent microplates to minimize sample handling. However, the microplates suitable for measuring both UV–vis absorbance and fluorescence and thus a very simple application of eq 1 for the IFE correction are considerably more expensive than non-transparent microplates. To estimate the general applicability of the ZINFE method, which does not require absorbance measurements, all measurements were duplicated using another type of non-transparent microplate as a potentially cost-saving solution.

The proposed approach can be readily applied to virtually any analyte, provided that the appropriate movement of the optical element (or microplate) in the *z*-axis can be achieved in order to obtain at least two measurements with different *z*-positions. As far as we know, this is the first attempt at IFE correction specifically intended for measurements in microplates.

## Experimental Section

IFE correction was first evaluated using a concentration series of a known fluorophore, quinine sulfate (QS), which was chosen as the reference analyte due to its frequent use in similar studies (concentration series Q).^8^ In order to test for both pIFe and sIFE, additional experiments were performed for different concentration series of QS in the presence of potassium dichromate (PD), which is known to absorb light at both the excitation and emission wavelengths of QS without exhibiting fluorescence itself.^14^ Specifically, PD was added to the QS concentration series: (i) at a fixed ratio of total concentrations of PD and QS in order to observe the behavior of the proposed IFE correction in the presence of an additional proportional background absorbance at the excitation wavelength (i.e., variable total concentrations of QS and PD; concentration series Q-v) or (ii) at a fixed total concentration of PD in order to observe the behavior of the proposed IFE correction in the presence of an additional constant background absorbance (i.e., variable ratio of total concentrations of QS and PD; concentration series Q-f).^13^ This was done because the samples may contain either a fixed or a proportional amount of additional absorber(s) in the working solutions (e.g., reaction buffer and storage buffer, respectively).

All experiments were performed at room temperature. The concentration range for the measurement was chosen to correspond to a maximum total absorbance at the excitation wavelength of *A*_ex_ ≈ 2, which is acceptable for most spectrophotometers and should be common in most experimental setups. In the experiments with added PD, the concentrations were chosen so that the maximum concentration of PD corresponds to *A*_ex_ ≈ 1. Full details on reagents and sample preparation can be found in Section 2 of the Supporting Information.

All measurements were performed in triplicate. The IFE corrections were performed using the averaged values of the background-corrected triplicate fluorescence and absorbance measurements (Section 4, Figures S8 and S11, Supporting Information). Separate calculations were also performed for data without background correction. All experiments were performed in parallel with two different types of microplates. The UV-transparent microplates (black, 96-well, μ-clear, flat bottom, chimney well, cat. no. 655097, Greiner, USA) allowed measurements of both absorbance and fluorescence intensity. The non-transparent microplates (black, 96-well, flat bottom, cat. no. 30122298, Tecan, Austria) allowed measurements of fluorescence intensity only.

For absorbance IFE corrections, a total of 9 corrections (eq 1) were obtained for each concentration series, corresponding to a separate IFE-corrected data set for each different *z*-position. For the *z*-position IFE corrections (ZINFE, eq 5), the measured fluorescence intensity values (*F*_1_) obtained for each *z*-position were corrected using the fluorescence intensity values (*F*_2_) obtained for the remaining *z*-positions. A total of *n* (*n* – 1) = 72 corrections were obtained. As a measure of linearity, the *R*^2^ statistic was calculated for each data set. The *z*-position correction whose *R*^2^ value was closest to 1 was selected as optimal and used to compare the results.^12,13^

For the NINFE correction (eq 6), the exponential term *N* corresponding to the optimal combination of positions *z*_1_ and *z*_2_ found by the procedure described above was chosen as the starting point (seed) for numerical optimization. This starting point is then varied in a series of 20 steps with a step size of 1 in both the positive and negative directions to produce a series of *R*^2^ values. An exponent corresponding to the maximum *R*^2^ value is then used as the seeding point in the next optimization cycle with the same number of steps in both directions, while the step size is decreased by a factor of 10. This procedure continues for 10 cycles or when the difference between the exponents from successive cycles is Δ*N* < 1 × 10^–6^, whichever comes first.

**Figure 2:**
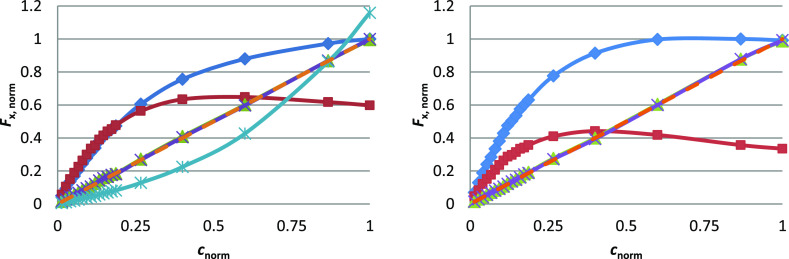
Results of the ZINFE correction: left: Q concentration series in UV-transparent microplates (data set 1); right: Q-v concentration series in non-transparent microplates (data set 4); *F*_1_ (blue diamond solid), *F*_2_ (brown box solid), *F*_Z_ (green triangle up solid), *F*_N_ (purple multiplication), *F*_A_ (blue asterisk), and IFS (orange hyphen). Ordinate values were calculated as *F*_x,norm_, and abscissa values were calculated as *c*_norm_. All results can be found in Figure S9, Supporting Information.

For all comparisons shown in Figure 2 and Table 1, the original and absorbance-corrected data correspond to the *z*-position (*z*_1_) used for the best *z*-position correction. Therefore, for each concentration series in a given microplate, all values are derived from the same value of *F*_1_ (corresponding to the uncorrected data) used in eqs 1, 5, and 6. For data processing, a dedicated script was written in the Javascript programming language.^15^ Full details on background correction and other data processing, including statistical considerations, can be found in the Supporting Information, Section 3.

**Table 1:** Overview of the Least-Squares Linear Fit Results for Normalized, Background-corrected Fluorescence and Absorbance Data

sample^a^	plate type^b^	correction type^c^	*R*^2^	*b* %^d^	LOD %^e^	*z*_1_/mm	Δ*z*^f^/mm	*c*_max_^g^/μM	*A*_max_^h^ (λ_ex_, λ_em_)
Q	T (data set 1)	*F*_1_	0.87449	17.5	36.4	19.0	2.0	679.3	1.984, 0.158
		*F*_Z_	0.99980	0.54	1.39				
		*F*_N_	0.99984	0.24	1.20				
		*F*_A_	0.95074	–7.87	21.9				
	NT (data set 2)	*F*_1_	0.81861	21.7	45.2	18.0	2.5		
		*F*_Z_	0.99971	0.12	1.64				
		*F*_N_	0.99973	–0.08	1.59				
Q-v	T (data set 3)	*F*_1_	0.81967	21.3	45.1	19.0	2.0	316.0	1.873, 0.443
		*F*_Z_	0.99951	0.95	2.13				
		*F*_N_	0.99964	0.43	1.83				
		*F*_A_	0.93753	–8.15	24.8				
	NT (data set 4)	*F*_1_	0.73752	25.9	57.3	18.0	2.0		
		*F*_Z_	0.99974	0.47	1.55				
		*F*_N_	0.99979	0.14	1.38				
Q-f	T (data set 5)	*F*_1_	0.98744	5.39	10.8	18.0	1.0	312.9	1.921, 0.464
		*F*_Z_	0.99959	–0.12	1.94				
		*F*_N_	0.99965	0.22	1.80				
		*F*_A_	0.98111	–4.85	13.3				
	NT (data set 6)	*F*_1_	0.98918	4.93	10.0	18.0	3.0		
		*F*_Z_	0.99964	1.24	1.83				
		*F*_N_	0.99972	0.89	1.61				

a Q corresponds to the pure QS concentration series; Q-v corresponds to the variable concentration of the absorber PD; Q-f corresponds to the fixed total concentration of PD.

b T corresponds to the UV-transparent microplates; NT corresponds to the non-transparent microplates. Data set numbers correspond to the averaged triplicate data preformatted for automated processing.^16^

c *F*_1_ corresponds to uncorrected fluorescence; *F*_Z_ corresponds to ZINFE-corrected fluorescence intensity (eq 5); *F*_A_ corresponds to absorbance IFE-corrected fluorescence intensity (eq 1); *F*_N_ corresponds to NINFE-corrected fluorescence intensity.

d Percent error of the normalized data slope with respect to the IFS. The values of slope and intercept used for data normalization for each concentration series are given in Table S12, Supporting Information.

e LOD (α = β = 0.05); the values were normalized as percentage of *c*_max_.

f Defined as Δ*z* = *z*_2_ – *z*_1_, where *z*_1_ and *z*_2_ are the different *z*-positions used for measurements of *F*_1_ and *F*_2_ (eq 5).

g Maximum concentration of QS in the concentration series.

h Maximum absorbance at the excitation and emission wavelengths, *λ*_ex_ = 345 nm and *λ*_em_ = 390 nm, respectively.

For further evaluation of the method and for immediate availability, an online service was set up to run the full correction algorithm at https://ninfe.science.^15^ All averaged triplicate data preformatted for automatic online processing and the results obtained have been archived.^16^

## Results and Discussion

The results of the ZINFE correction for the Q concentration series in UV-transparent microplates are shown in Figure 2. The values of *F*_1_ and *F*_2_ deviate from linearity due to IFE caused by increasing sample concentration. Although both *F*_1_ and *F*_2_ are recorded for the same samples in the same microplate, they are measured at different *z*-positions, resulting in different sample geometries and different dependences of the measured fluorescence on sample concentration. However, the values of *F*_1_ and *F*_2_ obtained in this way can be used to calculate the corrected *F* with improved linearity according to eqs 5 or 6. The corresponding results for all concentration series can be found in Figure S9, Supporting Information.

For convenient comparison of all results, the data were normalized as follows: (i) abscissa values were calculated as *F*_x,norm_ = *A*_ex_/*A*_max_, where *A*_ex_ is the baseline-corrected absorbance at the excitation wavelength and *A*_max_ is the maximum value of *A*_ex_ for the given concentration range; (ii) ordinate values were calculated as *c*_norm_ = *F*_x_/(*a* × *c*_max_ + *b*), where *F*_x_ corresponds to either the uncorrected or corrected fluorescence (*F*_1_, *F*_Z_, *F*_N_ or *F*_A_) and *a* and *b* are the slope and intercept, respectively, of the linear regression line for the corresponding data (Table S12, Supporting Information). The normalized values are 0 < *c*_norm_ < 1 and 0 < *F*_x,norm_ < ≈ 1, with maximum *F*_x,norm_ values depending on the deviation of the normalized value of *F*_A_, *F*_Z_, or *F*_N_ compared with the slope of the ideal fluorescence signal (IFS).

The IFS corresponds to the linear relationship between *F* and *A* in the absence of IFE.^17,18^ The slope of this linear relationship depends on the structural characteristics of the fluorophore, and the intercept should be equal to 0 after accounting for background fluorescence and absorbance *via* blank subtraction.^19^ Therefore, the value of IFS for the normalized data (i.e., plots of *F*_x,norm_ vs *c*_norm_) is a line with slope *a* = 1 and intercept *b* = 0, which allows very easy comparison of the uncorrected or corrected data with the ideal measurement response. A better match of the normalized data with the IFS requires a smaller deviation of the slope and the intercept of the linear regression from the values *a* = 1 and *b* = 0, respectively.

Considering the fact that *a* + *b* = 1 is valid for all normalized data, the value of *b* was given as a suitable measure of linearity and accuracy for comparing the different correction methods (Table 1). The values of *b* can be either positive or negative, corresponding to the downward or upward curvature of the fluorescence signal, respectively. In addition, the value of *b* obtained by the described normalization is numerically equal but opposite in sign to the percent error of the slope of the line of corrected fluorescence (*mErr* %, eq S7, Supporting Information), which was used by Gu and Kenny to compare IFE corrections.^13^ Therefore, the values of *b* were also expressed as *b* %, which means the percent error of the slope of the normalized data from IFS.

Another more conventional measure of linearity and accuracy of calibration curves is the limit of detection (LOD, eq S5, Supporting Information). The LOD value is defined as the concentration corresponding to an instrument signal for which the probability of false positive error (α) or false negative error (β) is a selected threshold percentage (in this study, α = β = 0.05).^20,21^ The LOD value appears to be particularly convenient because it contains both the measure of calibration sensitivity (i.e., the slope of the linear regression) and accuracy (i.e., the standard error of the estimate, *s*_*y*_, defined in eq S2, Supporting Information). For convenient comparison of the results, the LOD values obtained for the raw data (Table S12, Supporting Information) were normalized as a percentage of the highest concentration of the analyte in the corresponding series (*c*_max_), resulting in LOD % values (Table 1).

### Uncorrected Fluorescence (*F*_1_)

**Figure 3:**
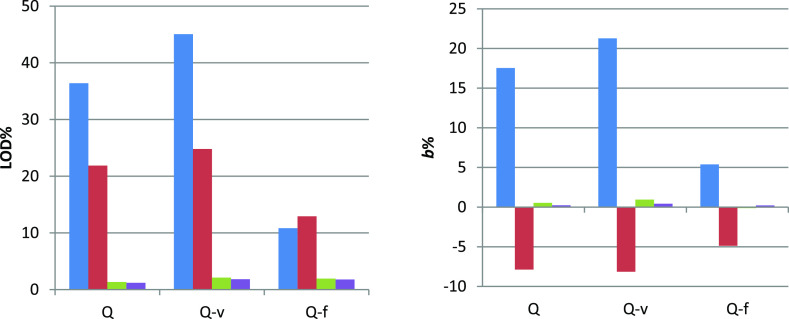
Comparison of uncorrected fluorescence (*F*_1_) and IFE-corrected fluorescence (*F*_A_, *F*_Z_, and *F*_N_) in UV-transparent microplates: left: LOD % from Table 1: *F*_1_ (blue box solid), *F*_A_ (red box solid), *F*_Z_ (green box solid), and *F*_N_ (violet box solid); right: *b* % from Table 1: *F*_1_ (blue box solid), *F*_A_ (red box solid) *F*_Z_ (green box solid), and *F*_N_ (violet box solid). Data are shown only for UV-transparent microplates and the data for non-transparent microplates are shown in Figures S19 and S20, Supporting Information.

The uncorrected data (*F*_1_) for the QS concentration series show a clear deviation from linearity, except for the Q-f concentration series, that is, for a fixed total concentration of PD (Figure S8, Supporting Information). The linearity of the uncorrected data depends on the *z*-position at which the fluorescence intensity was measured for all QS concentration series and increases with *z*-position (Figure S14, Supporting Information). The best *R*^2^ values are observed at *z* = 21 mm for all concentration series, which is consistent with increasing linearity of the fluorescence signal as the light path length decreases (higher *z*-values correspond to shorter light path lengths), that is, lower effective absorbance and thus smaller IFE. All deviations from the ideal signal were positive (i.e., *b* > 0, Figure 3, right), corresponding to a downward curvature for all concentration series due to IFE.

Linear regression of the uncorrected data at the *z*-position corresponding to the *z*_1_ value for the best ZINFE correction yield values of *R*^2^ < 0.875 and large values of LOD % > 36% of *c*_max_ and *b* % > 17%, consistent with the observed downward curvature of the fluorescence signal (Table 1, Q and Q-v concentration series). The Q-f concentration series gave slightly better results (*R*^2^ > 0.98, LOD % ≈ 10% of *c*_max_ and *b* % ≈ 5%), consistent with the observed lower curvature of the fluorescence signal compared with the Q and Q-v concentration series. The Q-f concentration series also showed the lowest dependence of *R*^2^ values on *z*-position (0.971 < *R*^2^ < 0.995, Figure S14, Supporting Information). Similar results for uncorrected data were also obtained for non-transparent microplates (all results can be found in Tables 1 and S12, Supporting Information). This observation can most likely be attributed to lower variability in total absorbance at the excitation wavelength for this concentration series compared to others (Figure S11 and Table S22, Supporting Information).

### Absorbance IFE-corrected Fluorescence (*F*_A_)

A total of 9 data sets per concentration series corresponding to different *z*-positions were obtained for UV-transparent microplates only (measured absorbance data can be seen in Figure S11, Supporting Information). Each absorbance IFE correction (*F*_A_, eq 1) gave better linearity than the uncorrected data, except for the Q-f concentration series (Table 1).

The linearity of the absorbance-corrected data also depends on the *z*-position at which fluorescence intensity was measured for all concentration series and decreases with *z*-position (Figure S15, Supporting Information). The variation in *R*^2^ values is smaller and also inverse to the dependence observed for uncorrected data (Figure S14, Supporting Information). This observation is consistent with increasing linearity of the absorbance-corrected fluorescence signal with increasing light path length (lower *z*-values correspond to longer light path lengths); that is, the effective absorbance approaches the value used for the correction. Notably, the correction factor in eq 2, (*A*_ex_ + *A*_em_)/2, is independent of *z*-position.

The best absorbance IFE corrections gave values *R*^2^ ≈ 0.99 and LOD % ≈ 10%, giving a linear response over approximately 90% of the concentration range with less than 3.5% deviation of the calibration slope from the ideal signal. All deviations from the ideal signal were negative (i.e., *b* < 0, Figure 3, right), corresponding to an upward curvature for all concentration series due to overcorrection (i.e., overestimated fluorescence loss) associated with the Lakowicz model, especially at higher absorbance.^4^ The Q-f concentration series again showed the least dependence of *R*^2^ values on *z*-position (0.975 < *R*^2^ < 0.989, Figure S15, Supporting Information). The absorbance IFE correction decreases the LOD % values by approximately 40%, compared to the uncorrected data for the Q and Q-v concentration series. Surprisingly, the LOD % value was increased by 20% compared to the uncorrected data for the Q-f concentration series, indicating that this type of correction is not appropriate in the presence of a background absorber.

### ZINFE-corrected Fluorescence (*F*_Z_)

A total of 72 data sets per concentration series were obtained, corresponding to different combinations of *z*-positions. The optimal *z*-position IFE correction (*F*_Z_, eq 5) significantly improves the linearity of the fluorescence signal for all QS concentration series, yielding values of *R*^2^ > 0.999 and deviation from the ideal signal response in the range of −0.122 < *b* % < 1.243. The LOD % values for all concentration series were in the range of 1.358–2.130% of the *c*_max_. Therefore, a linear response was obtained for all concentration series over approximately 98% of the concentration range with a maximum deviation of the calibration slope from the ideal signal of approximately 1% (Figure 3). For comparison, the uncorrected data at the same *z*-position for the entire concentration range gave values of *R*^2^ < 0.9, except for the Q-f series, which gave values of *R*^2^ < 0.99. The deviations from the ideal signal were much worse for the uncorrected data (*b* % ≈ 20% for the Q and Q-v concentration series, and *b* % ≈ 5% for the Q-f concentration series) and also for the absorbance-corrected values (*b* % ≈ −5%).

The quality of the *z*-position correction depends largely on the choice of *F*_1_ and *F*_2_ (i.e., the measured fluorescence values at different *z*-positions) used in eq 5. However, each ZINFE correction gave better linearity than the uncorrected data, and the best overall *R*^2^ value is obtained with the *z*-position correction. The three-dimensional plots for the dependence of the linear regression model error, calculated as Δ*R* = −1/(1 – *R*^2^), on the values of *z*_1_ and *z*_2_ showed a complex surface with multiple minima for all concentration series (Figure S13, Supporting Information). Such a shape of the error surface seems to justify the attempt of further numerical optimization according to eq 6.

### NINFE-corrected Fluorescence (*F*_N_)

The results obtained by numerical optimization of the exponent in eq 6 for a particular combination of *k*, *z*_1_, and *z*_2_, which yielded the highest *R*^2^ value, show a slight improvement compared with the calculation using geometry-dependent parameters (Table S16, Supporting Information). The exponents obtained from the geometric parameters and numerical optimization are in good agreement for Q and Q-v concentration series, with relatively small differences between the exponents (approximately 0.05), whereas slightly larger differences were obtained for the Q-f concentration series (approximately 0.2) (Table S16, Supporting Information). In general, the exponent optimization curves (Figure S17, Supporting Information) show remarkable similarity between the calculated and numerically optimized exponent values.

Regardless of the values of the differences in the exponents, similar improvements in the IFE correction were obtained for all concentration series: the *R*^2^ values were increased in the fourth or fifth decimal range, while the LOD % and *b* % were improved by approximately 0.5%, except for a single data set (*b* % was larger for Q-f concentration series in UV-transparent microplates).

### Transparent Versus Non-transparent Microplates and the Effect of Background Correction

**Figure 4:**
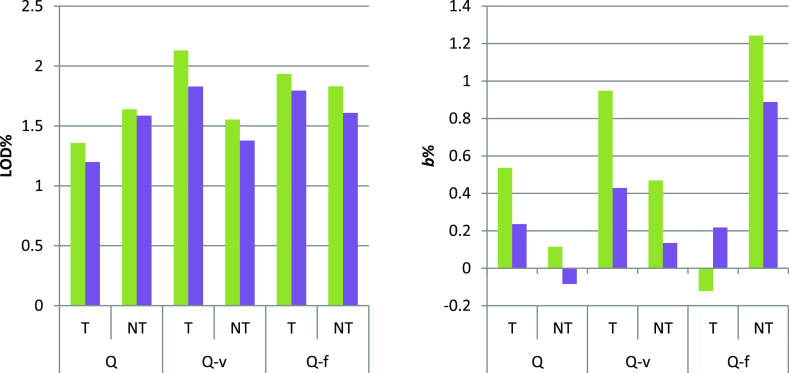
Comparison of ZINFE and NINFE corrections (*F*_Z_ and *F*_N_) in UV-transparent (T) and non-transparent (NT) microplates. Left: LOD % from Table 1: *F*_Z_ (green box solid) and *F*_N_ (violet box solid); right: *b* % from Table 1: *F*_Z_ (green box solid) and *F*_N_ (violet box solid).

The ZINFE and NINFE corrections performed in the two different types of 96-well plates gave very similar results. As can be seen in Figure 4, the LOD % and *b* % values for all *F*_Z_ corrections were comparable for all concentration series, with slightly better values obtained by numerical optimization (*F*_N_). A particularly interesting feature of the ZINFE correction or the NINFE correction is the ability to use fluorescence data without background correction. The results obtained for such data gave only slightly worse results, again with values of *R*^2^ > 0.999 for all concentration series with approximately 0.5% higher values of LOD and 0.4% higher absolute values of *b* %, compared with the data with background correction (Table S18 and Figures S19 and S20, Supporting Information). However, data without background correction should be used with caution because different behaviors of the background signal can be expected for samples other than those described here.

### IFE Correction for Low-Concentration Samples

Although the IFE correction may be considered unnecessary for low sample concentrations, we tested the use of this method for a lower range of sample concentrations. The uncorrected fluorescence (*F*_1_) is very linear (*R*^2^ > 0.994) for the first seven points in each concentration series. However, even for this concentration range, slightly increased *R*^2^ values and lower *b* % values were observed for the ZINFE- and NINFE-corrected data for the Q and Q-v concentration series in both UV-transparent and non-transparent microplates (Table S21, Supporting Information). Slightly decreased *R*^2^ values and higher *b* % values were observed for the Q-f concentration series for all IFE corrections, which may be attributed to increased noise due to the use of two measured values instead of only one. This is an indication that the regression residuals at low sample concentrations are mainly due to measurement errors rather than IFE.

## Conclusions

The described method of ZINFE correction is successful in extending the concentration range of the linear fluorescence signal for all concentration series, increasing the maximum applicable sample absorbance and eliminating the need for sample dilutions. The method is suitable for simultaneous correction of both pIFE and sIFE with an applicable maximum sample absorbance of at least *A*_ex_ ≈ 2 and *A*_em_ ≈ 0.5, with possible applicability at higher absorbance values. A simple heuristic for performing the measurements is to select a set of available *z*-positions depending on the characteristics of the microplate reader and find the optimal combination of *z*_1_ and *z*_2_ based on the quality of the linearization. In general, for this particular experimental setup, the best combinations of *z*-positions yielding the highest *R*^2^ values were obtained with *F*_1_ values measured at *z*_1_ = 18 or *z*_1_ = 19, while the *F*_2_ values are measured at 1–3 mm lower values of *z*_2_ (lower *z*-values correspond to a longer light path length).

Overall, the best corrections were obtained by numerical optimization of the exponent in eq 6. Thus, it was shown that the described method for NINFE correction provides an efficient IFE correction in microplates. The method does not require direct measurements of sample absorbance at the excitation and emission wavelengths or any additional parameters other than two fluorescence measurements at two different distances from the optical element of the microplate reader to obtain an IFE correction with a very linear response. The improvements obtained with NINFE could most likely be due to possible reflections from the walls of the microplate wells, which cannot be easily accounted for by geometric parameters alone. A major advantage of such numerical optimization is that no geometric parameters are needed, including the actual *z*-positions for the measurements. Moreover, NINFE can be used not only for measurements in microplate readers but also for any measurements obtained by the cell shift method. However, this method can be considered as a black-box system that may not be suitable for all users, who may then prefer to use the ZINFE method with known geometric parameters.

Both the ZINFE and NINFE methods give similar results compared to IFE corrections obtained with conventional spectrofluorometers. Lutz and Luisi, in their original work on the cell shift method, reported an accuracy of 3% for experiments with QS.^10^ Gu and Kenny have reported an accuracy of about 1.5% for their experiments with QS, while additional numerical optimization yielded an accuracy of about 0.2%.^13^ More recently, Panigrahi and Mishra reported an accuracy of about 0.5% for their experiments with QS (calculated from the values in the Supporting Information provided with the article).^4^ The results reported here are in good agreement with these values (|*b* %| < 1.3% for all concentration series, Figure 4). In addition, we have shown that both ZINFE and NINFE are comparably effective for samples with an additional absorber in varying proportions. Similarly, we have shown that both methods are comparably effective in both UV-transparent and non-transparent microplates. The extended linear response of the fluorescence signal provided by ZINFE or NINFE allows simplified fluorescence measurements without sample dilution, thus eliminating the often complex and time- and resource-consuming liquid handling associated with microplates.

## Supporting Information Available

Additional experimental details including instrumental parameters, sample preparation, statistical considerations, and results for all data sets (PDF)
