# Supplementary material for: Inner Filter Effect Correction for Fluorescence Measurements in Microplates Using Variable Vertical Axis Focus
Source: Anal Chem. 2022 May 3;94(19):7107–14. doi: 10.1021/acs.analchem.2c01031 (PMC9118198; doi:10.1021/acs.analchem.2c01031)
Supplement: Supplementary file 1 — ac2c01031_si_001.pdf [file ac2c01031_si_001.pdf]

Supplementary information

Inner filter effect correction for fluorescence measurements in microplates using variable vertical axis focus

Tin Weitner*, Tomislav Friganović, Davor Šakić

Faculty of Pharmacy and Biochemistry, University of Zagreb, Ante Kovačića 1, 10000 Zagreb, Croatia

Corresponding author:

Tin Weitner, PhD

e-mail: tin.weitner@pharma.unizg.hr

tel.: +385 1 6394 452

Contents

1. Instrumental parameters 3
2. Sample preparation 6
  2.1. Reagents 6
  2.2. General remarks 6
  2.3. Solutions 6
    a) 0.05 M sulfuric acid 6
    b) Quinine sulfate (QS) in 0.05 M sulfuric acid (concentration series Q) 6
    c) Quinine sulfate (QS) in 0.05 M sulfuric acid (concentration series Q-f and Q-v). 6
    d) Potassium dichromate (PD) in 0.05 M sulfuric acid (concentration series Q-f and Q-v) 7
    e) Spectral measurements 7
    f) Sample concentrations 9
3. Statistical considerations 10
  3.1. Quality of fit and linearity measures 10
    a) Coefficient of determination, R^2 10
    b) Standard error of the estimate, s_y 10
    c) Limit of detection, LOD 10
    d) Percent error of the slope, *mErr*% 11
  3.2. Error estimation 11
    a) Error estimation for absorbance (A), uncorrected fluorescence intensity (F_1 and F_2) and light path length (h) 11
    b) Error estimation for the exponential term (N) 12
    c) Error propagation for the absorbance IFE correction (F_A) 13
    d) Error propagation for the *z*-position IFE correction (ZINFE, F_Z) 14
4. Experimental data 16
  4.1. Fluorescence data 16
  4.2. Absorbance data 20
  4.3. Results of the linear regression for raw (unscaled) data. 21
  4.4. Error surfaces 22
  4.5. Miscellaneous information 23
Conflicts of interest 29
Acknowledgements 29
References 30

1. Instrumental parameters

Fluorescence and absorbance measurements were performed using the Tecan Spark M10 multimode microplate reader (Tecan, Austria). Fluorescence intensity was measured for λ_{ex} = 345 nm and λ_{em} = 390 nm using *z*-position values in the range of 14.6 - 21.0 mm (Table S1). The absorbance values for both wavelengths were measured to obtain the values A_{ex} and A_{em} (Eq. 1), respectively in UV-transparent microplates (Figure S11). Instrument settings of the microplate reader can be found in Table S3, SI. The solution volume in each microplate well was 200 µL. The distance from the bottom of the microplate well to the surface of the liquid, *h*, (Figure 1) was estimated for Greiner microplates by measuring the absorbance of pure water. The values of *h* for Tecan plates were measured using transparent microplates of the same geometry (transparent, 96-well, flat bottom, cat. no. 30122304, Tecan, Austria), allowing a correct calculation of *k* = 20.593 mm, which was used in calculations. Full details of the measurement of parameter *h* and specific values of geometric parameters in Figure 1 and Eq. 3, can be found in Table S2. Required geometric parameters of the microplate reader sample compartment and optical element were kindly provided by the manufacturer.

Table S1. Values of *z*-positions used for fluorescence intensity measurements and subsequent IFE corrections. The values are given in mm for clarity, and the actual instrumental parameter is adjustable to the nearest µm.

***z*-position number**	**z / mm**
1	14.6
2	15.0
3	15.5
4	16.0
5	17.0
6	18.0
7	19.0
8	20.0
9	21.0

Table S2. Values of geometric parameters shown in Figure 1 used for the *z*-position IFE corrections.

parameter	value / mm	significance
d	10.9	microplate well depth; value according to manufacturer's specifications
h	5.093 ± 0.042	distance from the bottom of the microplate well to the surface of the liquid; measurement described in the SI, Section 3.2
t	14.4	height of the microplate; value according to manufacturer's specifications
f	16	distance from the optical element to the focal point of the lens; value obtained from manufacturer specifications
m	4	depth of the optical element lens slot; value according to manufacturer's specifications
k	20.593	overall geometric parameter k for a particular experimental setup (i.e., sample volume, microplate, and microplate reader type), calculated using Eq. 4 (see Manuscript)

Table S3. Printout of the device settings for the Tecan Spark M10 multimode microplate reader.

Device:	Tecan Spark M10	
Application:	SparkControl V2.3	
Shaking (Linear) Duration	5	s
Shaking (Linear) Position	Current	
Shaking (Linear) Amplitude	1	mm
Shaking (Linear) Frequency	1440	rpm
**Mode**	**Fluorescence Top Reading**	
Excitation	Monochromator	
Excitation wavelength	345	nm
Excitation bandwidth	20	nm
Emission	Monochromator	
Emission wavelength	390	nm
Emission bandwidth	20	nm
Gain	40	Manual
Mirror	Automatic (50% Mirror)	
Number of flashes	30	
Integration time	40	µs
Lag time	0	µs
Settle time	0	ms
Z-Position mode	Manual	
**Mode**	**Absorbance**	
Wavelength start	200	nm
Wavelength end	700	nm
Wavelength step size	1	nm
Number of flashes	1	
Settle time	50	ms

Measurements were performed at ambient temperature (range 22.0 – 28.4 ºC, measured in the sample compartment of the microplate reader). The maximum temperature deviation for any concentration series was 0.18 ºC.

2. Sample preparation

2.1. Reagents

Water used for sample preparation was double distilled in an all-glass apparatus. Quinine sulfate (QS, 99.0-101.0 %, cat. no. 22640, Sigma-Aldrich, USA), potassium dichromate (PD, 99 %, cat. no. 1112907, Kemika, Croatia) and concentrated sulfuric acid (96 %, cat. no. 410261, Carlo Erba Reagents, France) were used without further purification. All experiments were performed in 0.05 M H_2SO_4 prepared from concentrated solution. All working solutions were prepared using Opentrons OT-2 liquid handling robot (Opentrons, USA).

2.2. General remarks

All titrations in all experiments were performed by pipetting into 0.65 mL centrifuge tubes. Liquid handling robot Opentrons OT-2 (Opentrons, USA) was used to prepare all samples from stock solutions. Volumes of less than or equal to 30 µL were dispensed into a larger volume of solution and then 30 µL of the solution was aspirated and dispensed again to rinse the tip. After pipetting, all tubes were capped and thoroughly mixed on a vortex mixer. After mixing, aliquots of 200 µL were transferred to microplates for measurement, again using the robot. Prior to measurement, microplates were centrifuged for 2 min at 2550 rpm using a microplate centrifuge (Benchmark Scientific, USA) with additional shaking in the microplate reader sample compartment for 5 s at 1440 rpm and amplitude of 1 mm.

2.3. Solutions

a) 0.05 M sulfuric acid

For all solutions containing QS and PD, 0.05 M sulfuric acid was used as solvent (details of chemicals are given in the manuscript). A solution of 0.05 M sulfuric acid was obtained by diluting concentrated sulfuric acid in an appropriate volume of redistilled water. The required volume of concentrated acid was calculated using the density and percent content indicated by the manufacturer on the original bottle.

b) Quinine sulfate (QS) in 0.05 M sulfuric acid (concentration series Q)

The QS stock solution was prepared by first dissolving an arbitrary amount of QS in 0.05 M sulfuric acid. The resulting solution has a very high absorbance, so aliquots of this solution were added to 0.05 M sulfuric acid to obtain the maximum absorbance $A_{ex} \sim 2$ in the concentration series. The actual concentration of QS for each point was calculated in triplicate from the absorbance measurements.

c) Quinine sulfate (QS) in 0.05 M sulfuric acid (concentration series Q-f and Q-v).

The procedure was the same as for the Q concentration series, except that the maximum absorbance of the QS was $A_{ex} \sim 1$ in the concentration series Q-f and Q-v.

d) Potassium dichromate (PD) in 0.05 M sulfuric acid (concentration series Q-f and Q-v)

The PD stock solution was prepared by first dissolving an arbitrary amount of PD in 0.05 M sulfuric acid and diluting it so that the maximum absorbance of the stock solution was A_{ex} ~ 5. For experiments with fixed total concentration of PD, aliquots of this stock solution were added to obtain the constant absorbance A_{ex} ~ 1 for PD in all samples in the Q-f concentration series. For experiments with variable total concentration of PD, different aliquots of this stock solution and 0.05 M sulfuric acid were added to obtain increasing absorbance to the maximum of A_{ex} ~ 1 for PD in the Q-v concentration series.

e) Spectral measurements

UV/Vis spectra were measured using Varian Cary 50 spectrophotometer (Varian, Australia) in a quartz cuvette (l = 1 cm) at room temperature. Values for QS were normalized to the reference value of ε_{345} = 5700 M^{-1} cm^{-1} in 0.05 M sulfuric acid and are given in Table S4.[1] Values for PD were normalized to the reference value of ε_{350} = 3150 M^{-1} cm^{-1} in 0.01 M sulfuric acid.[2] Briefly, the spectrum of PD was measured in 0.05 M H_2SO_4, the solution was diluted 5 times with water to give 0.01 M H_2SO_4 and the spectrum was measured again. For each solution, the corresponding background (solution of 0.05 M H_2SO_4 and the same solution diluted 5 times with water) was measured and subtracted from the spectrum of PD. The value of ε_{350} in 0.05 M H_2SO_4 was calculated as (A_{350} (0.05 M H_2SO_4) / (A_{350} (0.01 M H_2SO_4) × 5)) × 3150 M^{-1} cm^{-1} = 2965 M^{-1} cm^{-1}. The values of ε_{345} and ε_{390} were then calculated from the ratio of the measured absorbance relative to A_{350} and are given in Table S4.

The fluorescence spectrum of QS was measured at room temperature using Olis RSM 1000F spectrofluorometer (Olis, USA). The excitation wavelength was 345 nm ($A_{345} \approx 1$) and the excitation bandwidth was 13 nm. The fluorescence units (f.u.) correspond to the ratio of signals obtained from sample and reference PMTs. The fluorescence spectrum was normalized to the maximum value obtained at 452 nm.

Table S4. Molar absorbance coefficients, ε, for quinine sulfate and potassium dichromate at excitation and emission wavelengths, λ_{ex} = 345 nm and λ_{em} = 390 nm, respectively.

**Sample**	ε_{345} **/ M^{-1} cm^{-1}**	ε_{390} **/ M^{-1} cm^{-1}**
Quinine sulfate	5700	348
Potassium dichromate	2939	1049

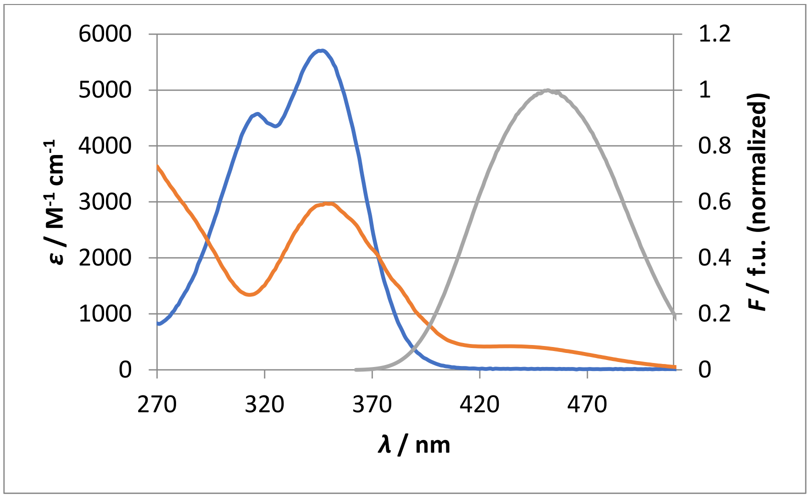

Figure S5. Molar absorbance spectra for quinine sulfate (—) and potassium dichromate (—) in 0.05 M H_2SO_4 at room temperature. The secondary *y*-axis is used for the normalized fluorescence spectrum of quinine sulfate (—).

f) Sample concentrations

A total of 3 concentration series were prepared in triplicate and each concentration series was measured in 2 types of microplates (transparent or non-transparent), corresponding to a total of 6 datasets:

Dataset 1: concentration series Q in UV-transparent microplates
Dataset 2: concentration series Q in non-transparent microplates
Dataset 3: concentration series Q-v in UV-transparent microplates
Dataset 4: concentration series Q-v in non-transparent microplates
Dataset 5: concentration series Q-f in UV-transparent microplates
Dataset 6: concentration series Q-f in non-transparent microplates

Separate calculations were performed with and without background correction (see manuscript).

Table S6. Sample concentrations of quinine sulfate and potassium dichromate for all concentration series; Q, Q-v and Q-f.

Sample number	*c* / µM					ratio [QS]/[PD]	
	Quinine Sulfate (QS)			Potassium dichromate (PD)			
	Q [1]	Q-v [1]	Q-f [1]	Q-v [1]	Q-f [1]	Q-v [1]	Q-f [1]
1	9.057	4.214	4.172	8.173			0.006
2	18.11	8.428	8.344	16.35			0.013
3	27.17	12.64	12.52	24.52			0.019
4	36.23	16.86	16.69	32.69			0.025
5	45.28	21.07	20.86	40.86			0.032
6	54.34	25.28	25.03	49.04			0.038
7	63.40	29.50	29.20	57.21			0.044
8	72.45	33.71	33.38	65.38			0.051
9	81.51	37.92	37.55	73.55			0.057
10	90.57	42.14	41.72	81.73	658.7	0.516	0.063
11	99.62	46.35	45.89	89.90			0.070
12	108.7	50.57	50.06	98.07			0.076
13	117.7	54.78	54.24	106.2			0.082
14	126.8	58.99	58.41	114.4			0.089
15	181.1	84.28	83.44	163.5			0.127
16	271.7	126.4	125.2	245.2			0.190
17	407.6	189.6	187.7	367.8			0.285
18	588.7	273.9	271.2	531.2			0.412
19	679.3	316.0	312.9	613.0			0.475

[1]Q corresponds to the pure QS concentration series; Q-v corresponds to the variable concentration of the absorber PD (fixed ratio of the total concentrations of PD and QS); Q-f corresponds to the fixed total concentration of PD (variable ratio of the total concentrations of PD and QS), see manuscript for details.

3. Statistical considerations

3.1. Quality of fit and linearity measures

a) Coefficient of determination, R^2

This represents the proportion of the variance of the dependent variable that is explained by the independent variable(s) in a regression model, and is defined as:[3]

$$R^2 = \frac{(\mathrm{cov}(x,y))^2}{\mathrm{var}(x)\mathrm{var}(y)} = \frac{(\mathrm{SS}_{xy})^2}{\mathrm{SS}_{xx}\mathrm{SS}_{yy}}. \qquad \text{Eq S1}$$

Values closer to 1 indicate a better fit.

b) Standard error of the estimate, s_y

This represents the measure of variation used to check the accuracy of the predictions made with the regression line, and is defined as:[4]

$$s_y = \sqrt{\frac{1}{(n-2)}\left[\sum_{i=1}^{n}(y_i - \bar{y})^2 - \frac{\left[\sum_{i=1}^{n}(x_i-\bar{x})(y_i-\bar{y})\right]^2}{\sum_{i=1}^{n}(x_i-\bar{x})^2}\right]}, \qquad \text{Eq. S2}$$

where n is the number of data points for linear interpolation. Values closer to 0 indicate a better fit.

c) Limit of detection, LOD

This is defined as the least amount of a substance that can be distinguished from the blank (i.e. absence of the substance) at a given confidence level, i.e. probability of false positive error (α) or false negative error (β).

The background-corrected signal, $y_{\mathrm{SAMPLE}} - y_{\mathrm{BLANK}}$, is proportional to the sample concentration c:

$$y_{\mathrm{SAMPLE}} - y_{\mathrm{BLANK}} = m \cdot c, \qquad \text{Eq. S3}$$

where y_{BLANK} is the signal from the blank sample and m is the slope of the calibration line.

Limit of detection is then defined as:[5]

$$\mathrm{LOD} = \frac{n s_y}{m}, \qquad \text{Eq. S4}$$

where s_y is the standard error of the estimate (eq. S2) and n is chosen depending on the confidence level required.

For a chosen confidence level of 5 % (i.e., $\alpha = \beta = 0.05$), the eq. S5 amounts to:[3,6]

$$\mathrm{LOD} = \frac{3.3s}{m}. \quad \text{Eq. S5}$$

d) Percent error of the slope, *mErr%*

Originally calculated from the slope *m* of the line of corrected fluorescence (LCF), compared to the slope of the line of dilute solutions (LDS), defined as:[7]

$$\mathrm{mErr}\ \% = (m_{\mathrm{LCF}} - m_{\mathrm{LDS}})/m_{\mathrm{LDS}} \cdot 100\ \%. \quad \text{Eq. S6}$$

Considering that the ideal fluorescence signal, IFS, which corresponds to the linear relationship between *F* and *A* in the absence of IFE, is a line with slope $a = 1$ and intercept $b = 0$ for normalized data, the eq. S3 simplifies to:[3,8,9]

$$\mathrm{mErr}\ \% = (a - 1)/1 \cdot 100\ \%. \quad \text{Eq. S7}$$

where *a* is the slope of the linear regression line for normalized data (Table 1 in the manuscript). Values closer to 0 indicate a better fit.

3.2. Error estimation

a) Error estimation for absorbance (*A*), uncorrected fluorescence intensity (F_1 and F_2) and light path length (*h*)

The sample standard deviations, *s*, were estimated for all absorbance and fluorescence intensity measurements (denoted as x_i) as shown in equation:

$$s = \sqrt{\frac{1}{n-1}\sum_{i=1}^{n}(x_i - \bar{x})^2}. \quad \text{Eq. S8}$$

The sample variances were calculated using the equation:

$$\mathrm{var}(x) = s^2 = \frac{1}{n-1}\sum_{i=1}^{n}(x_i - \bar{x})^2. \quad \text{Eq. S9}$$

Background corrections were performed for all absorbance and fluorescence intensity measurements. Values measured at zero concentrations of the fluorophore were subtracted from each data point in the fluorophore concentration series. The variance of each background-corrected data point (x_{BC}) was calculated as the sum of the data point (x) variance and the background (x_{B}) variance:

$$\mathrm{var}(x_{\mathrm{BC}}) = \mathrm{var}(x) + \mathrm{var}(x_{\mathrm{B}}). \quad \text{Eq. S10}$$

We have assumed that there is no correlation between the background and the errors of the titration data points. The main source of variability is likely due to the pipetting errors, which are expected to be random and uncorrelated.

Standard deviations were calculated for the background-corrected data as follows:

$$s = \sqrt{\mathrm{var}(x_{\mathrm{BC}})} = \sqrt{\mathrm{var}(x) + \mathrm{var}(x_{\mathrm{B}})}. \qquad \text{Eq. S11}$$

For aqueous solutions, the path length can be calculated from the absorbance values for water in the near-infrared wavelength range (900 nm to 1000 nm) using a cuvette and the corresponding microplate. The estimation of h was made by measuring the path length of pure water with parameters: test wavelength λ = 977 nm, reference wavelength λ = 900 nm and correction factor value of 0.186. The correction factor is defined as the absorbance value of water at the test wavelength corrected by the absorbance value of water at the reference wavelength for a path length of 1 cm.[10] The path length of pure water was measured in decaplicate and the average value of the path length obtained for a sample volume of 200 µL was used as the h-estimator.

b) Error estimation for the exponential term (*N*)

The exponential coefficient in eqs. 5 and 6 in the manuscript can be written as*:*

$$N = \frac{l_1}{l_1 - l_2} = \frac{d-(h-t)+f-z_2}{d-(h-t)+f-z_1-(d-(h-t)+f-z_2)} = \frac{d-(h-t)+f-z_2}{z_2-z_1}. \qquad \text{Eq. S12}$$

The uncertainties in the geometric parameters f and z were not considered in the error estimation calculations. We did not have numerical values for these uncertainties, which result from the tolerances in the manufacture of microplates. The Tecan Spark M10 multimode microplate reader software displays the parameter z in 5 significant figures. The parameter f is a spatial dimension that should be easily measured with high precision.

It is reasonable to assume that the variations of these parameters are statistically insignificant compared to the variation of the parameter h caused by pipetting errors. Therefore, the standard deviation of the term N is calculated to be equal to the standard deviation of the parameter h, assuming that the standard deviations of the parameters f, z_1, and z_2 are statistically insignificant:

$$s(N) \approx s(h). \qquad \text{Eq. S13}$$

The values of $s(N)$ were calculated only for combinations of z-positions that gave the best results in the correction procedure and were used for comparison with uncorrected (F_1) and absorbance-corrected fluorescence (F_{A}).

c) Error propagation for the absorbance IFE correction (F_A)

The correction function given in eq. 1 is a function of three variables and can be written as follows:

$$f(F_1,\ A_{ex},\ A_{em}) = F_1 \cdot 10^{[(A_{ex} + A_{em})/2]}. \qquad \text{Eq. S14}$$

The partial derivatives of the above function were calculated with respect to all three variables as:

$$\frac{\partial f(F_1,\ A_{ex},\ A_{em})}{\partial F_1} = 10^{[(A_{ex} + A_{em})/2]}, \qquad \text{Eq. S15}$$

$$\frac{\partial f(F_1,\ A_{ex},\ A_{em})}{\partial A_{ex}} = F_1 \cdot \ln(10) \cdot 2^{\left[\frac{1}{2}(A_{ex} + A_{em} - 2)\right]} \cdot 5^{\left[\frac{1}{2}(A_{ex} + A_{em})\right]}, \qquad \text{Eq. S16}$$

$$\frac{\partial f(F_1,\ A_{ex},\ A_{em})}{\partial A_{em}} = \frac{\partial f(F_1,\ A_{ex},\ A_{em})}{\partial\ A_{ex}}. \qquad \text{Eq. S17}$$

The error propagation was estimated using the following expressions:[4]

$$s_f = \left(\frac{\partial f}{\partial F_1} \cdot s_{F_1}\right)^2 + \left(\frac{\partial f}{\partial A_{ex}} \cdot s_{A_{ex}}\right)^2 + \left(\frac{\partial f}{\partial A_{em}} \cdot s_{A_{em}}\right)^2, \qquad \text{Eq. S18}$$

$$s_f{}' = \left(\frac{\partial f}{\partial F_1} \cdot s_{F_1}\right)^2 + \left(\frac{\partial f}{\partial A_{ex}} \cdot s_{A_{ex}}\right)^2 + \left(\frac{\partial f}{\partial A_{em}} \cdot s_{A_{em}}\right)^2$$

$$+ 2 \cdot \left(\frac{\partial f}{\partial F_1} \cdot \frac{\partial f}{\partial A_{ex}}\right) \cdot \mathrm{cov}(F_1, A_{ex}) + 2 \cdot \left(\frac{\partial f}{\partial F_1} \cdot \frac{\partial f}{\partial A_{em}}\right) \cdot \mathrm{cov}(F_1, A_{em})$$

$$+ 2 \cdot \left(\frac{\partial f}{\partial A_{ex}} \cdot \frac{\partial f}{\partial A_{em}}\right) \cdot \mathrm{cov}(A_{ex}, A_{em}). \qquad \text{Eq. S19}$$

The first expression (eq. S18) is used to calculate the standard deviation for the F_A values without considering the covariance factors. The second expression (eq. S19) considers the covariance terms calculated for all the pairs of variables (F_1, A_{em}, A_{ex}). The covariance terms seem to be significant in the total sum, considering that it is reasonable to assume that errors in the three variables are not independent of each other, since they are probably the result of pipetting errors and/or geometric imperfections and/or contamination of the microplate. Therefore, eq. S19 was used to calculate the standard deviation for the F_A values.

d) Error propagation for the *z*-position IFE correction (ZINFE, F_Z)

The correction function given in eqs. 5 and S12 is a function of three variables, and can be written as follows:

$$g(F_1, F_2, N) = F_1 \left(\frac{F_1}{F_2}\right)^N. \qquad \text{Eq. S20}$$

The exponential term N is a constant for each IFE correction procedure, since it is a function of the pairs of z-position values (z_1 and z_2) used for the particular correction. The term N varies from one correction to another as the combinations of z-position pairs also vary in this respect.

The partial derivatives of the above function were calculated with respect to all three variables as:

$$\frac{\partial g(F_1,\ F_2,\ N)}{\partial F_1} = (N+1) \cdot \left(\frac{F_1}{F_2}\right)^N, \qquad \text{Eq. S21}$$

$$\frac{\partial g(F_1,\ F_2,\ N)}{\partial F_2} = -N \cdot \left(\frac{F_1}{F_2}\right)^{(1+N)}, \qquad \text{Eq. S22}$$

$$\frac{\partial g(F_1,\ F_2,\ N)}{\partial N} = F_1 \cdot \left(\frac{F_1}{F_2}\right)^N \cdot \ln\left(\frac{F_1}{F_2}\right). \qquad \text{Eq. S23}$$

The error propagation was estimated using the following expressions:

$$s_g = \left(\frac{\partial g}{\partial F_1} \cdot s_{F_1}\right)^2 + \left(\frac{\partial g}{\partial F_2} \cdot s_{F_2}\right)^2 + \left(\frac{\partial g}{\partial N} \cdot s_N\right)^2, \qquad \text{Eq. S24}$$

$$s_g' = \left(\frac{\partial g}{\partial F_1} \cdot s_{F_1}\right)^2 + \left(\frac{\partial g}{\partial F_2} \cdot s_{F_2}\right)^2 + \left(\frac{\partial g}{\partial N} \cdot s_N\right)^2 + \mathrm{cov}(F_1, F_2). \qquad \text{Eq. S25}$$

Similar to eqs. S18 and S19, the eq. S24 was used to calculate the standard deviation for the F_Z values without considering the covariance factors. The eq. S25 considers the covariance terms calculated for the F_1 and F_2 values (N is constant for each correction). The covariance term may also be significant in the overall sum, since it depends on the combination of z-positions used for a particular correction. Therefore, eq. S25 was used to calculate the standard deviation for the F_Z values.

In general, it can be observed that for the closest pairs of z-positions (in terms of numerical values) there is often a very significant correlation. This can be easily verified by plotting the F_1 vs. F_2 values (Figure S7). This shows a very good positive correlation between the measured F values over the entire concentration range for the combination of adjacent z-position measurements (z = 14.6 mm and z = 15 mm, red symbols). However, for the pair of the most distant z-positions (z = 14.6 mm and z = 21 mm, blue symbols), there is significantly worse correlation measured F values, especially at the highest concentrations of the fluorophore.

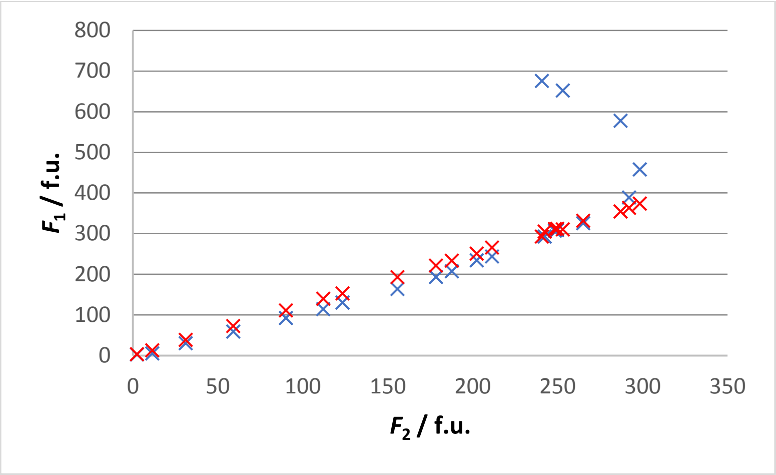

Figure S7. Plot of F_1 (z = 15 mm, ×, and z = 21 mm, ×) *vs*. F_2 (z = 14.6 mm) for the Q concentration series.

4. Experimental data

All averaged triplicate data preformatted for automatic online processing and the results obtained have been permanently archived.[11]

In total, there are 6 different datasets for 3 different concentration series (Q, Q-v and Q-f) in 2 different types of microplates (T and NT). For each concentration series, a total of 9 fluorescence measurements were performed using the selected available *z*-positions ($n = 9$, Table S1).

4.1. Fluorescence data

The measured data and the results of the ZINFE correction (F_Z) and the NINFE correction (F_N) are summarized in Figures S8, S9 and S10.

All plots in these figures were created using the JavaScript open-source graphing library Plotly[12] in the online calculator available at https://ninfe.science.[13]

Due to incompatible algorithms, two separate online calculators were created: (i) for the proposed ZINFE and NINFE correction, and (ii) for the absorbance IFE correction. The online service requires the properly formatted fluorescence measurements and *z*-position data (both for NINFE and ZINFE), as well as known geometric parameters for the specific microplate and microplate reader (for ZINFE only).

a) Q – transparent microplates (Dataset 1)

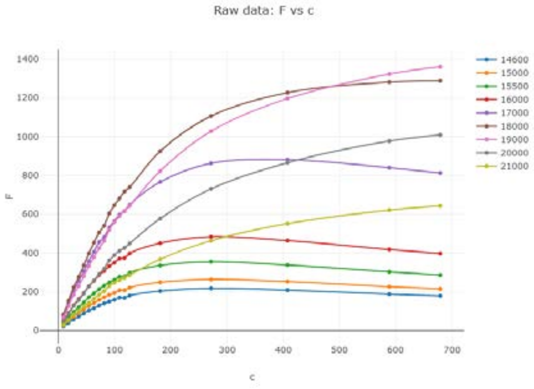

b) Q – non-transparent microplates (Dataset 2)

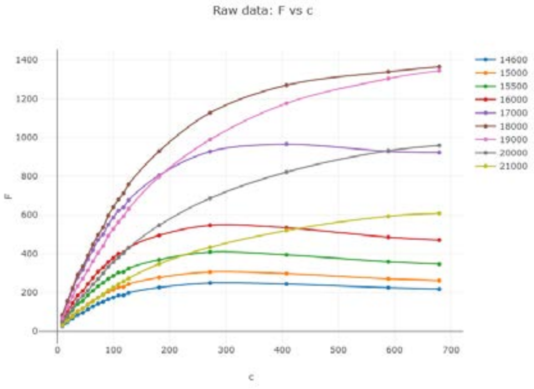

c) Q-v – transparent microplates (Dataset 3)

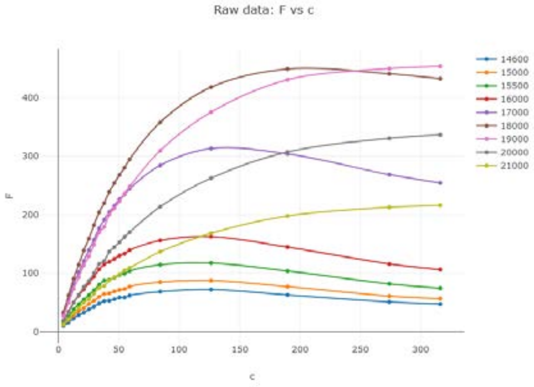

d) Q-v – non-transparent microplates (Dataset 4)

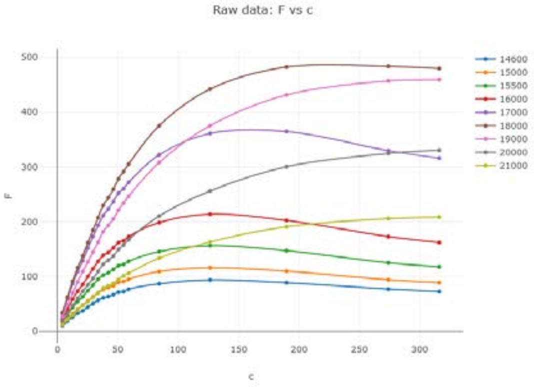

e) Q-f – transparent microplates (Dataset 5)

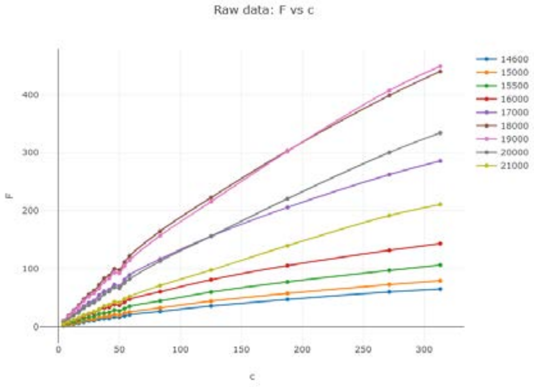

f) Q-f – non-transparent microplates (Dataset 6)

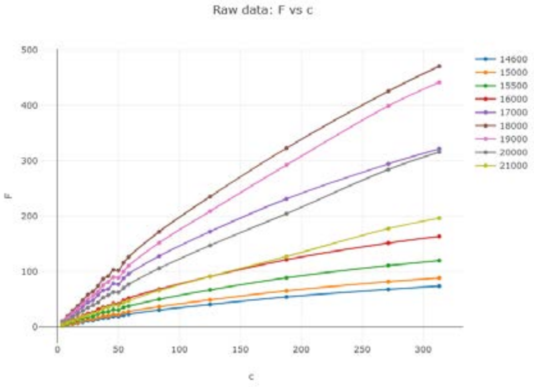

Figure S8. Dependence of background-corrected fluorescence measured at different *z*-positions on QS concentration (values of *z*-positions are given in the legend of each plot).

a) Q – transparent microplates (Dataset 1)

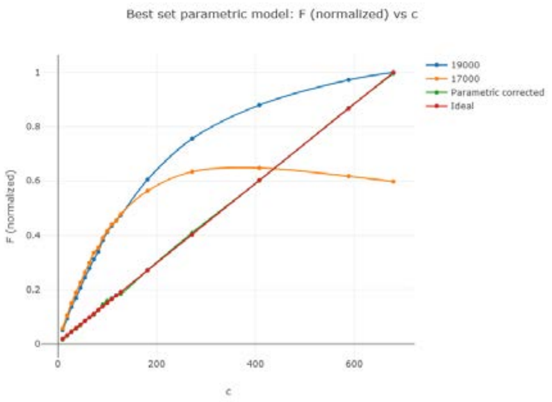

b) Q – non-transparent microplates (Dataset 2)

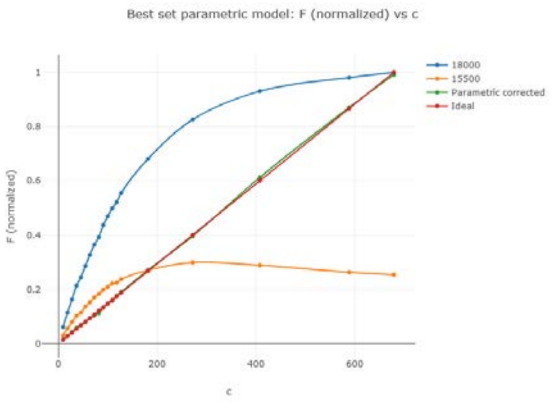

c) Q-v – transparent microplates (Dataset 3)

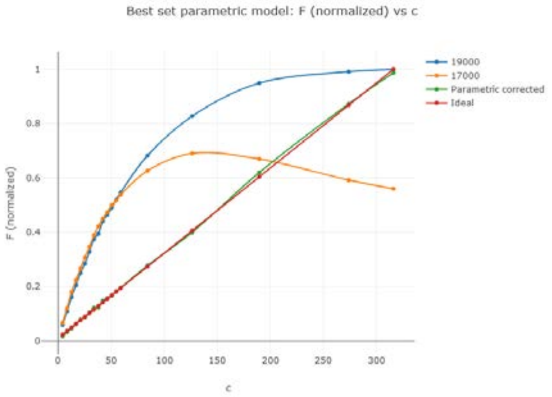

d) Q-v – non-transparent microplates (Dataset 4)

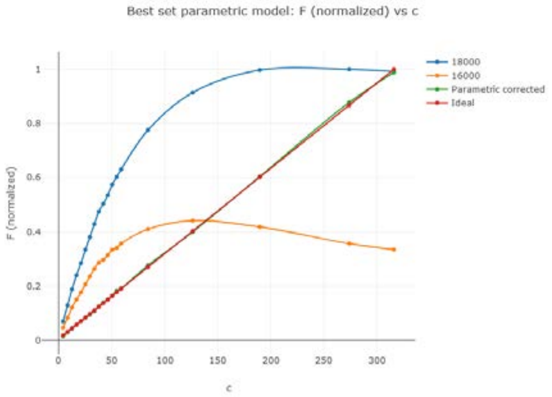

e) Q-f – transparent microplates (Dataset 5)

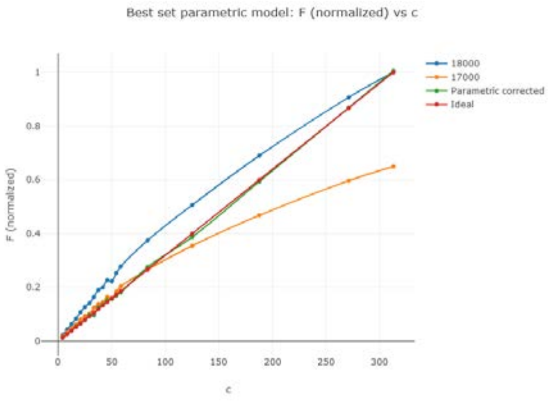

f) Q-f – non-transparent microplates (Dataset 6)

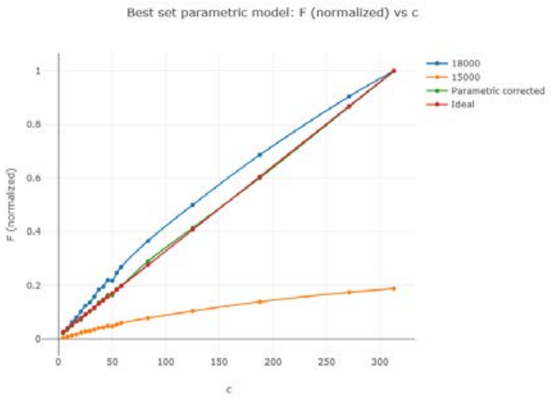

Figure S9. Comparison of the optimal ZINFE corrections (F_Z, —), the uncorrected values of (F_1, —) and (F_2, —) used for the calculation, and the ideal fluorescence signal (IFS, —). All values of F_x were normalized as described in the manuscript.

a) Q – transparent microplates (Dataset 1)

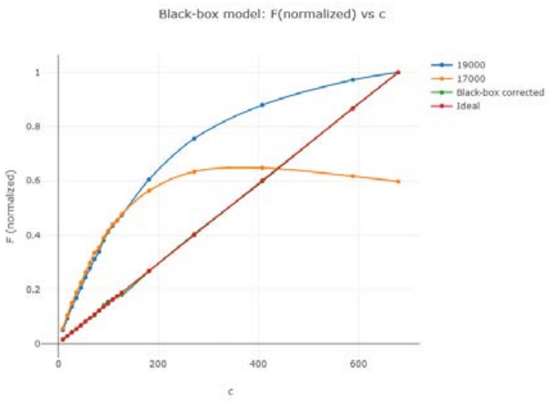

b) Q – non-transparent microplates (Dataset 2)

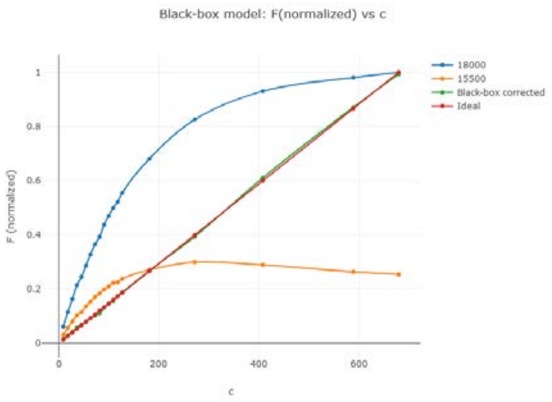

c) Q-v – transparent microplates (Dataset 3)

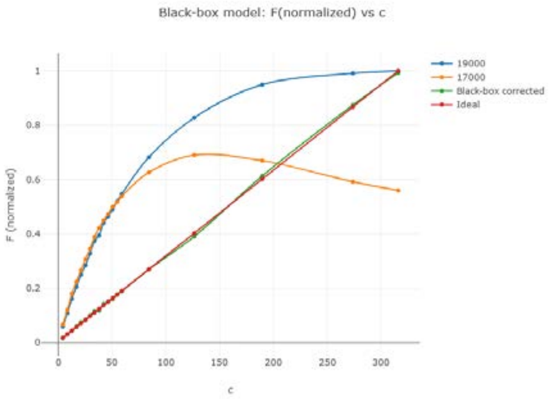

d) Q-v – non-transparent microplates (Dataset 4)

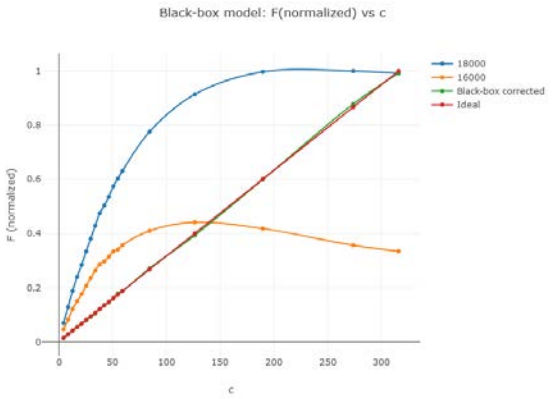

e) Q-f – transparent microplates (Dataset 5)

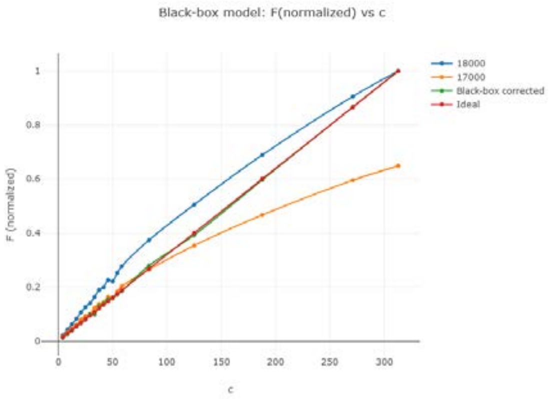

f) Q-f – non-transparent microplates (Dataset 6)

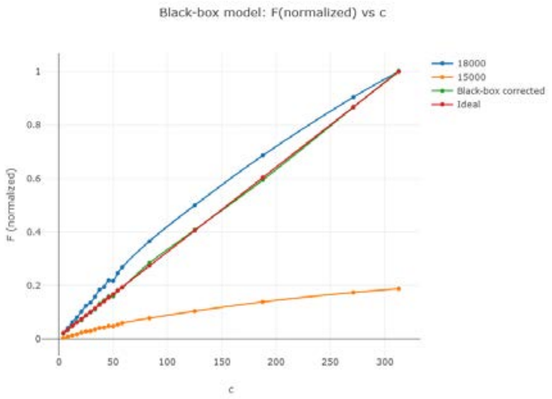

Figure S10. Comparison of the optimal NINFE corrections (F_N, —), the uncorrected values of (F_1, —) and (F_2, —) used for the calculation, and the ideal fluorescence signal (IFS, —). All values of F_x were normalized as described in the manuscript.

4.2. Absorbance data

a) Q concentration series (Dataset 1)

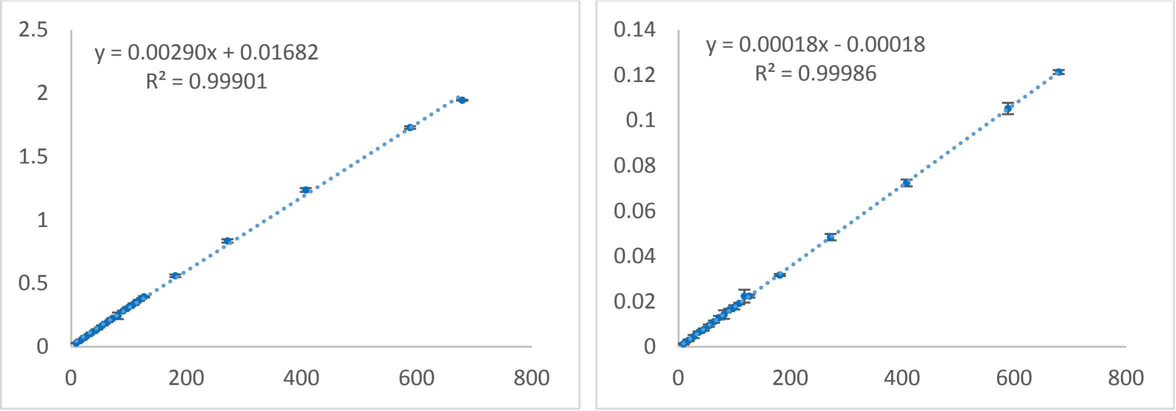

b) Q-v concentration series (Dataset 3)

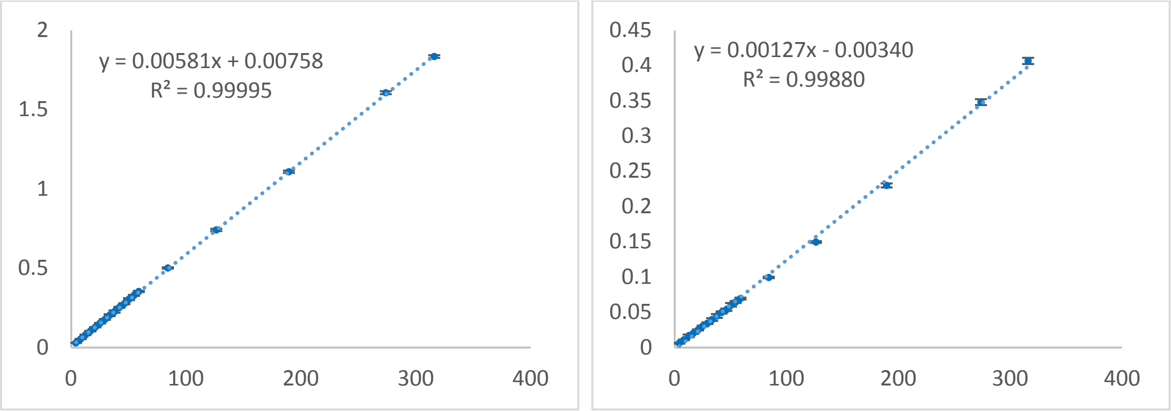

c) Q-f concentration series (Dataset 5)

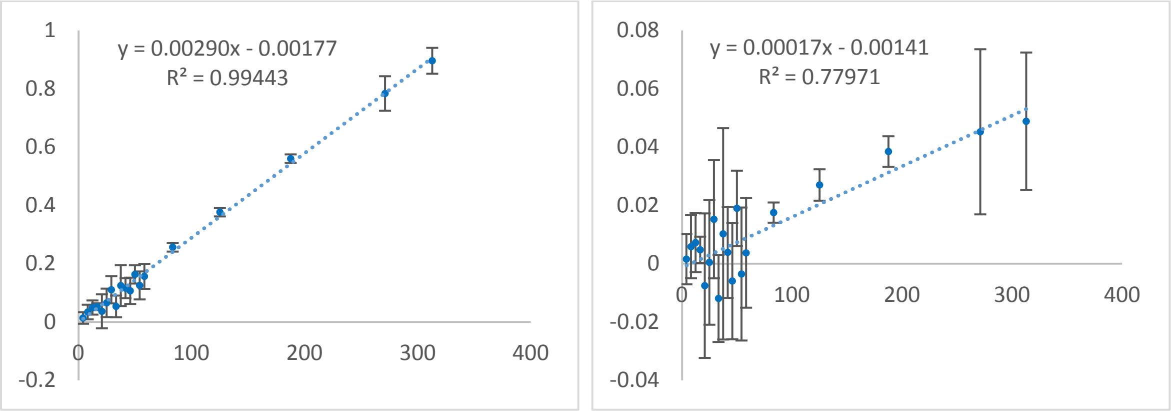

Figure S11. Dependence of the absorbance at excitation and emission wavelengths on QS concentration (UV-transparent microplates only): **left:** A_{ex} values; **right**: A_{em} values. The error bars denote standard deviations of the measurements in triplicate. The results of the linear regression are shown in the insets of individual plots.

4.3. Results of the linear regression for raw (unscaled) data.

Table S12. Overview of least squares linear fit results for unscaled, background-corrected data. Slope and intercept values were used for data normalization (Table 1 in the manuscript; see manuscript for details).

**Sample** [1]	**Plate type** [2]	**correction type**[3]	R^2	**Slope (*a*) / µM^{-1}**	**Intercept (*b*)**	s_y [5]	**LOD** [6] **/ µM**
Q	T	F_1	0.87449	1.9	276.6	143.5	247.2
		F_Z	0.99980	5.0	18.5	14.1	9.2
		F_N	0.99984	5.2	8.3	12.8	8.1
		F_A	0.95074	20.1	-997.3	906.2	148.5
	NT	F_1	0.81861	1.9	349.6	173.4	307.2
		F_Z	0.99971	33.0	25.9	111.5	11.1
		F_N	0.99973	34.5	-19.7	112.6	10.8
Q-v	T	F_1	0.81967	1.3	115.2	58.2	142.4
		F_Z	0.99951	4.1	12.4	8.3	6.7
		F_N	0.99964	4.3	5.8	7.5	5.8
		F_A	0.93753	17.2	-409.6	408.5	78.4
	NT	F_1	0.73752	1.4	152.0	75.7	181.1
		F_Z	0.99974	18.5	27.5	27.5	4.9
		F_N	0.99979	19.6	8.4	25.8	4.4
Q-h	T	F_1	0.98744	1.4	25.0	14.4	33.9
		F_Z	0.99959	6.6	-2.5	12.1	6.1
		F_N	0.99965	5.9	4.0	10.1	5.6
		F_A	0.98111	4.0	-57.8	50.5	41.7
	NT	F_1	0.98918	1.5	24.4	14.3	31.4
		F_Z	0.99964	33.4	131.6	58.0	5.7
		F_N	0.99972	46.7	131.0	71.3	5.0

[1] Q corresponds to the pure QS concentration series; Q-v corresponds to the variable concentration of the absorber PD (fixed ratio of the total concentrations of PD and QS); Q-f corresponds to the fixed total concentration of PD (variable ratio of the total concentrations of PD and QS), see manuscript for details.
[2] T corresponds to UV-transparent microplates; NT corresponds to non-transparent microplates.
[3] F_1 corresponds to uncorrected fluorescence; F_Z corresponds to ZINFE-corrected fluorescence intensity (eq. 5); F_A corresponds to absorbance IFE-corrected fluorescence intensity (eq. 1); F_N corresponds to NINFE-corrected fluorescence intensity.
[4] Standard error of the estimate defined by eq. S2.
[5] Limit of detection ($\alpha = \beta = 0.05$); see manuscript for details.
[6] LOD values normalized as percentage of c_{max}, see manuscript for details.

4.4. Error surfaces

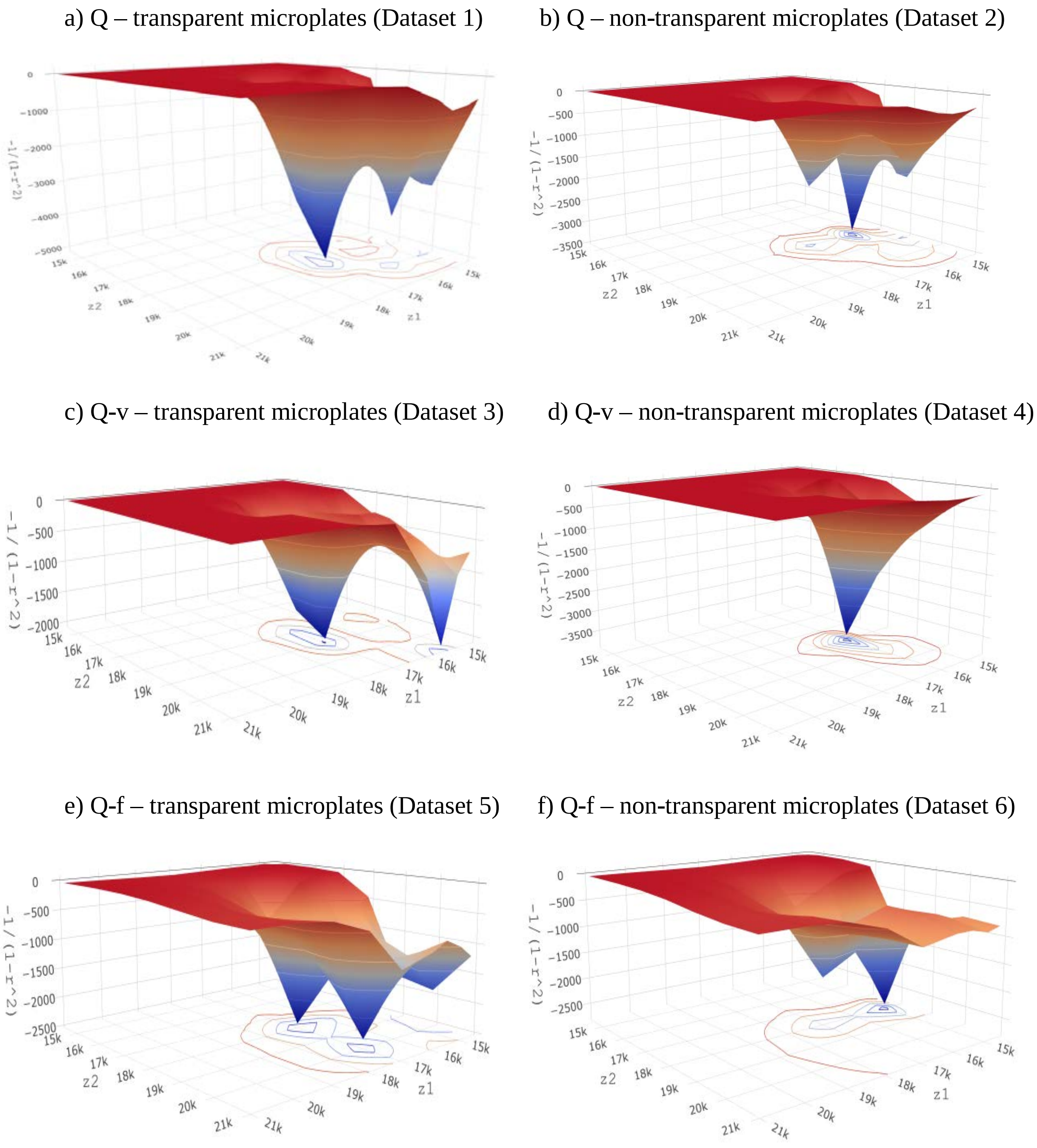

Figure S13. The 3D plot for the dependence of the model error, $\Delta R = -1 / (1 - R^2)$, on the values of z_1 and z_2. All plots were created using the JavaScript open-source graphing library Plotly[12] and the online calculator NINFE.[13]

4.5. Miscellaneous information

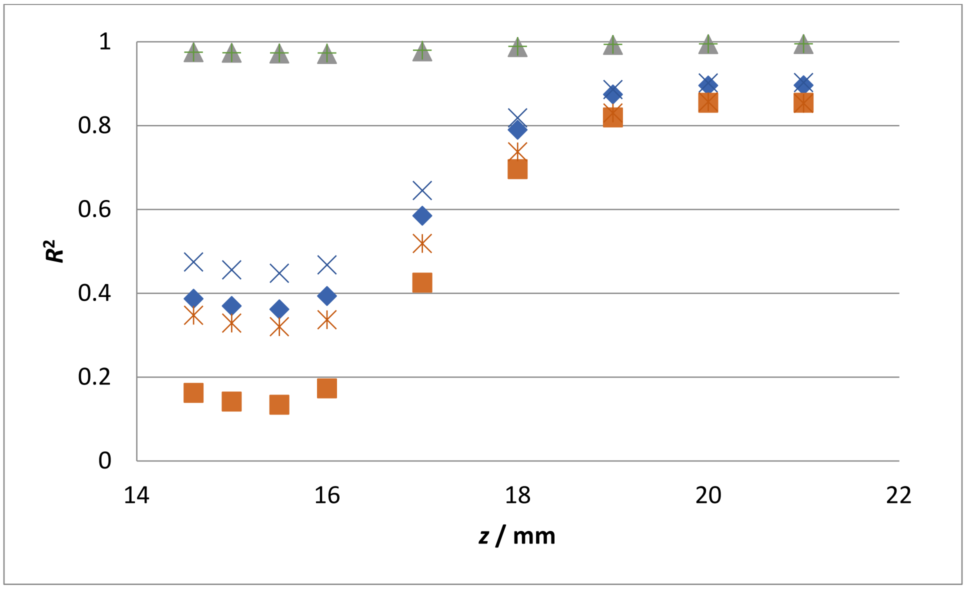

Figure S14. Dependence of calculated R^2 values for uncorrected fluorescence (F_1) on *z*-position: UV-transparent plates, concentration series: Q (◆), Q-v (■) and Q-f (▲); non-transparent plates, concentration series: Q (×), Q-v (✳) and Q-f (+).

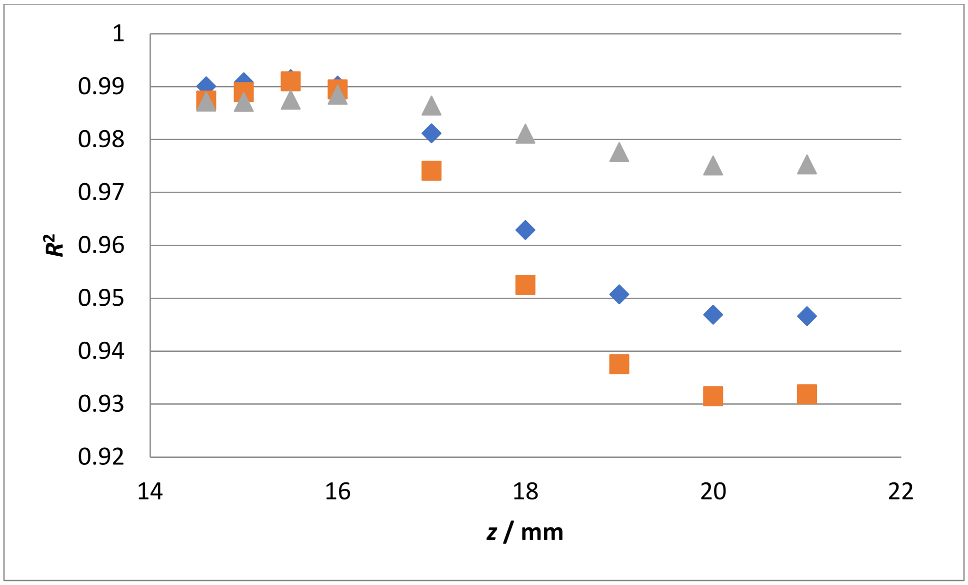

Figure S15. Dependence of calculated R^2 values for absorbance-corrected data (F_A) on *z*-position: concentration series Q (◆), Q-v (■) and Q-f (▲). Data are shown only for UV-transparent plates.

Table S16. Comparison of exponents resulting from geometric parameters (eq. 5) and numerical optimization (eq. 6).

**Sample** [1]	**Plate type** [2]	**Correction type** [3]	**Exponent *N***	$\|F_Z - F_N\|$
Q	T	F_Z	-1.7965	*0.0480*
		F_N	*-1.8445*	
	NT	F_Z	-2.0372	*0.0312*
		F_N	*-2.0684*	
Q-v	T	F_Z	-1.7965	*0.0730*
		F_N	*-1.8695*	
	NT	F_Z	-2.2965	*0.0522*
		F_N	*-2.3487*	
Q-f	T	F_Z	-3.593	*0.2600*
		F_N	*-3.333*	
	NT	F_Z	-1.8643	*0.2017*
		F_N	*-2.066*	

[1,2,3] See Table S12 and manuscript for details.

a) Q – transparent microplates (Dataset 1)

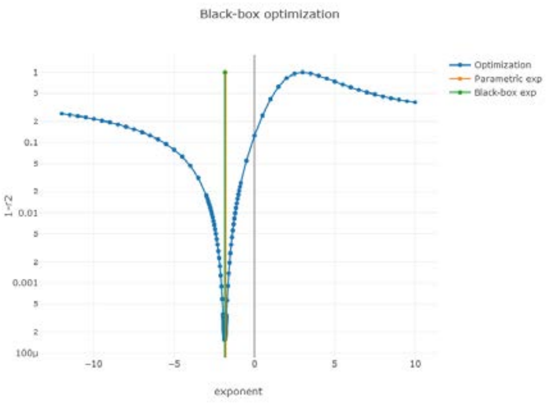

b) Q – non-transparent microplates (Dataset 2)

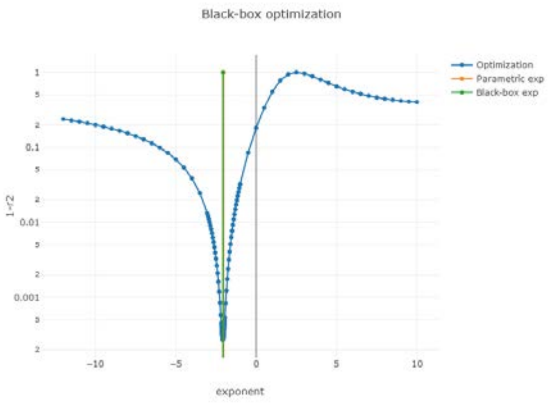

c) Q-v – transparent microplates (Dataset 3)

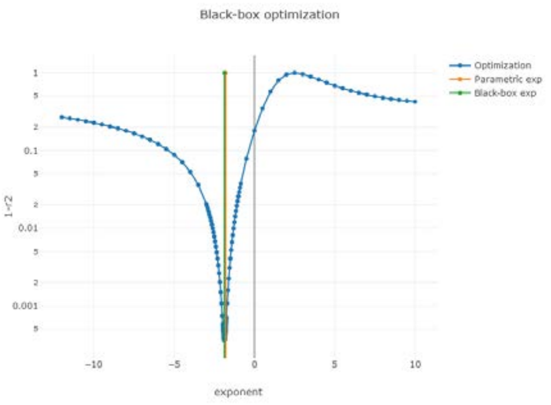

d) Q-v – non-transparent microplates (Dataset 4)

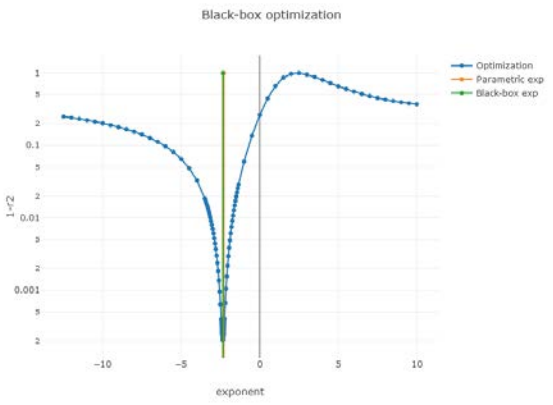

e) Q-f – transparent microplates (Dataset 5)

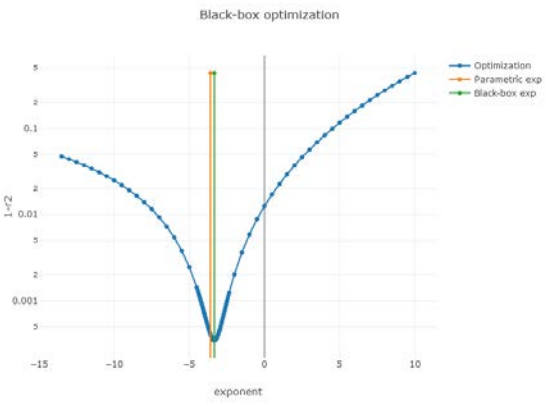

f) Q-f – non-transparent microplates (Dataset 6)

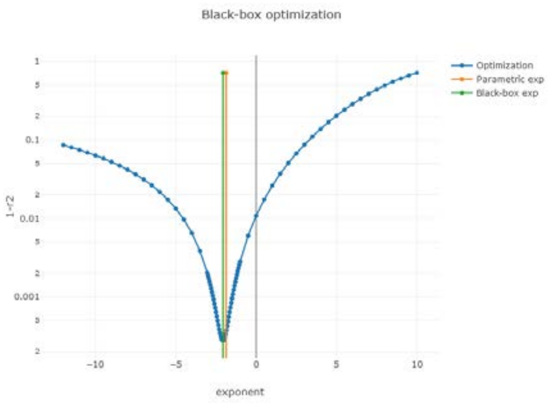

Figure S17. Exponent optimization error curves: exponent value used for ZINFE correction (F_Z, —), exponent value used for NINFE correction (F_N, —). All plots were created using JavaScript open-source graphing library Plotly[12] and the online calculator NINFE.[13]

Table S18. Comparison of linear regression of data with and without background correction (F_Z and F_N).

Sample [1]	Plate type [2]	Correction type [3]	WITH background correction [4]			WITHOUT background correction [5]		
			R^2	LOD%	*b* %	R^2	LOD%	*b* %
Q	T	F_Z	0.99964	1.82	0.68	0.99980	1.36	0.54
		F_N	0.99985	1.16	-0.01	0.99984	1.20	0.24
	NT	F_Z	0.99968	1.72	0.20	0.99971	1.64	0.12
		F_N	0.99972	1.60	-0.11	0.99973	1.59	-0.08
Q-v	T	F_Z	0.99924	2.65	-1.28	0.99951	2.13	0.95
		F_N	0.99925	2.63	-1.16	0.99964	1.83	0.43
	NT	F_Z	0.99957	1.99	0.76	0.99974	1.55	0.47
		F_N	0.99979	1.40	0.09	0.99979	1.38	0.14
Q-f	T	F_Z	0.99961	1.90	-0.16	0.99959	1.93	-0.12
		F_N	0.99967	1.74	0.22	0.99965	1.79	0.22
	NT	F_Z	0.99962	1.86	1.02	0.99964	1.83	1.24
		F_N	0.99972	1.59	0.57	0.99972	1.61	0.89

[1, 2, 3] See Table S12 and manuscript for details.
[4] Background-corrected data, copied from Table 1 in the manuscript for clarity.
[5] No background correction, i.e. only raw sample fluorescence data was used for IFE correction.

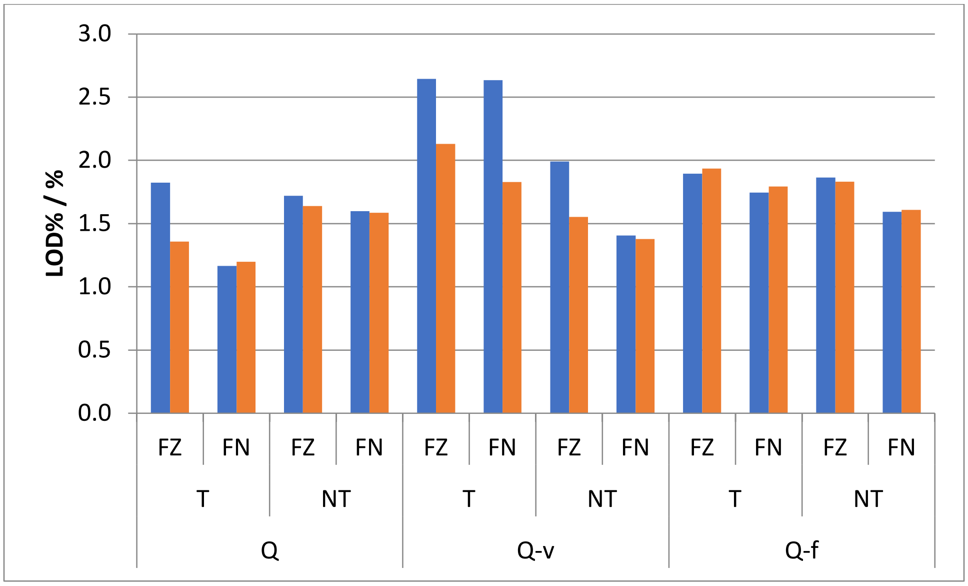

Figure S19. Comparison of LOD% values (F_Z and F_N): with background correction (■), without background correction (■). All values are LOD% < 2.7 % for ZINFE correction and LOD% < 2.2 % for NINFE correction.

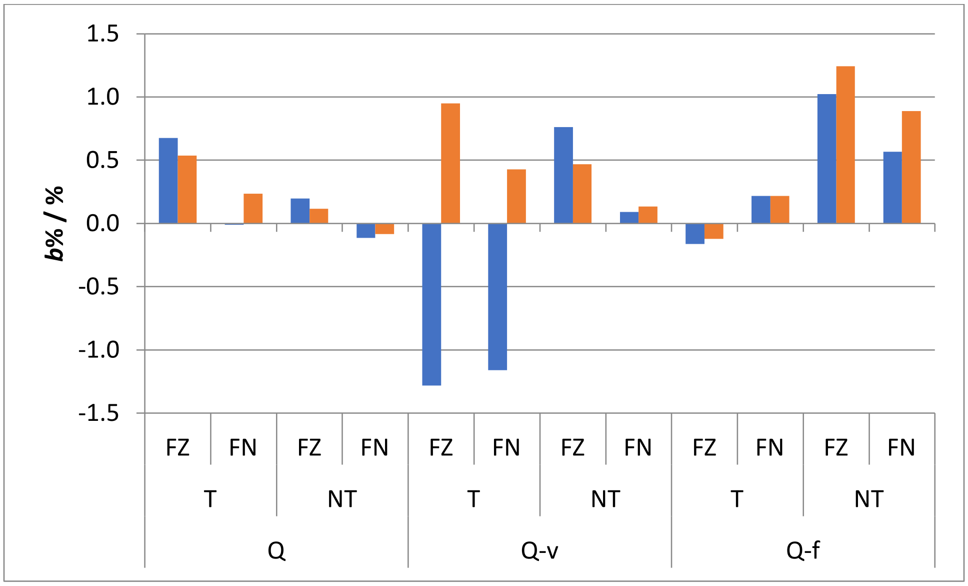

Figure S20. Comparison of *b* % values (F_Z and F_N): with background correction (■), without background correction (■). The absolute values of all *b* % values, corresponding to the direction-insensitive deviation from the ideal fluorescence signal, are |*b* %| < 1.3 % for either the ZINFE correction or the NINFE correction.

Table S21. Overview of the least-squares linear fit results for normalized, background-corrected fluorescence and absorbance data in the low concentration range (the first 7 points of each dataset).

Sample [1]	Plate type [2]	Correction type [3]	R^2	*b* %	LOD%
Q	T	F_1	0.9983	5.92	4.67
		F_Z	0.9986	2.31	4.11
		F_N	0.9986	2.22	4.15
		F_A	0.9988	-0.33	3.93
	NT	F_1	0.9944	7.95	8.37
		F_Z	0.9964	3.89	6.70
		F_N	0.9964	3.83	6.70
Q-v	T	F_1	0.9964	6.37	6.68
		F_Z	0.9968	2.96	6.32
		F_N	0.9967	2.83	6.41
		F_A	0.9996	0.17	2.25
	NT	F_1	0.9972	6.82	5.96
		F_Z	0.9988	2.69	3.91
		F_N	0.9988	2.60	3.88
Q-f	T	F_1	0.9973	2.25	5.78
		F_Z	0.9968	-2.68	6.37
		F_N	0.9975	-2.34	5.57
		F_A	0.9957	-1.09	7.31
	NT	F_1	0.9967	2.24	6.41
		F_Z	0.9946	7.76	8.25
		F_N	0.9931	8.42	9.28

[1, 2, 3] See Table S12 and manuscript for details.

Table S22. Overview of the total change of absorbance, ΔA, for all concentration series, calculated from the data shown in Figure S11.

Sample [1]	ΔA	
	**excitation**	**emission**
Q	1.92	0.12
Q-v	1.80	0.40
Q-f	0.88	0.04

[1,] See Table S12 for details.

Conflicts of interest

There are no conflicts of interest to declare.

Acknowledgements

This work was supported by funding from the Croatian Science Foundation grant UIP-2017-05-9537 – *Glycosylation as a factor in the iron transport mechanism of human serum transferrin* (GlyMech). Additional support was provided by the European Structural and Investment Funds for the 'Croatian National Centre of Research Excellence in Personalized Healthcare' (contract #KK.01.1.1.01.0010), 'Centre of Competences in Molecular Diagnostics' (contract #KK.01.2.2.03.0006), and the European Regional Development Fund grant for 'Strengthening of Scientific Research and Innovation Capacities of the Faculty of Pharmacy and Biochemistry at the University of Zagreb' (FarmInova, contract #KK.01.1.1.02.0021).

References

(1) Taniguchi, M.; Lindsey, J. S. Database of Absorption and Fluorescence Spectra of >300 Common Compounds for Use in PhotochemCAD. *Photochemistry and Photobiology* **2018**, *94* (2), 290–327. https://doi.org/10.1111/php.12860.

(2) Spijkerman, J. J.; Travis, J. C.; Pella, P. A.; DeVoe, J. R. Preliminary Study on the Characteristics and Design Parameters for Mossbauer Resonant Detector, 1971.

(3) Miller, J. N.; Miller, J. C. *Statistics and Chemometrics for Analytical Chemistry*, 6. ed.; Prentice Hall: Harlow, 2010.

(4) Taylor, J. R. *An Introduction to Error Analysis: The Study of Uncertainties in Physical Measurements*, 2nd ed.; University Science Books: Sausalito, Calif, 1997.

(5) ICH Harmonised Tripartite Guideline: Validation of Analytical Procedures: Text and Methodology Q2 (R1); Geneva, Switzerland, 2005; Vol. 11. https://doi.org/10.1163/ej.9789004163300.i-1081.897.

(6) Brunetti B, D. E. About Estimating the Limit of Detection by the Signal to Noise Approach. *Pharm Anal Acta* **2015**, *06* (04). https://doi.org/10.4172/2153-2435.1000355.

(7) Gu, Q.; Kenny, J. E. Improvement of Inner Filter Effect Correction Based on Determination of Effective Geometric Parameters Using a Conventional Fluorimeter. *Anal. Chem.* **2009**, *81* (1), 420–426. https://doi.org/10.1021/ac801676j.

(8) Holland, J. F.; Teets, R. E.; Kelly, P. M.; Timnick, Andrew. Correction of Right-Angle Fluorescence Measurements for the Absorption of Excitation Radiation. *Anal. Chem.* **1977**, *49* (6), 706–710. https://doi.org/10.1021/ac50014a011.

(9) Kothawala, D. N.; Murphy, K. R.; Stedmon, C. A.; Weyhenmeyer, G. A.; Tranvik, L. J. Inner Filter Correction of Dissolved Organic Matter Fluorescence. *Limnology and Oceanography: Methods* **2013**, *11* (12), 616–630. https://doi.org/10.4319/lom.2013.11.616.

(10) Tecan Spark Instructions for Use – Reference Guide V1.2, 2017.

(11) Weitner, T.; Friganović, T.; Šakić, D. Inner Filter Effect Correction for Fluorescence Measurements in Microplates, 2022. https://doi.org/10.5281/zenodo.5849302.

(12) Plotly Technologies Inc., C. Plotly JavaScript Graphing Library https://plotly.com/javascript/.

(13) Šakić, D.; Weitner, T.; Friganović, T. NINFE https://ninfe.science/.
